# Alleviating rheumatoid arthritis with a photo-pharmacotherapeutic glycan-integrated nanogel complex for advanced percutaneous delivery

**DOI:** 10.1186/s12951-024-02877-8

**Published:** 2024-10-21

**Authors:** Pei-Wei Weng, Hsien-Tsung Lu, Lekshmi Rethi, Chia-Hung Liu, Chin-Chean Wong, Lekha Rethi, Kevin C.-W. Wu, Pei-Ru Jheng, Hieu T. Nguyen, Andrew E.-Y. Chuang

**Affiliations:** 1 Department of Orthopedics, School of Medicine, College of Medicine, Taipei Medical University, 250 Wu-Hsing Street, Taipei, 11031 Taiwan; 2 Graduate Institute of Biomedical Materials and Tissue Engineering, Graduate Institute of Nanomedicine and Medical Engineering, College of Biomedical Engineering, Taipei Medical University, New Taipei City, Taiwan; 3 Department of Orthopedics, Shuang Ho Hospital, Taipei Medical University, New Taipei City, 23561 Taiwan; 4 Research Center of Biomedical Devices, Taipei Medical University, Taipei, 11031 Taiwan; 5 International Ph.D. Program for Cell Therapy and Regenerative Medicine, College of Medicine, Taipei Medical University, Taipei, 11031 Taiwan; 6 Department of Urology, School of Medicine, College of Medicine, Taipei Medical University, 250 Wu-Hsing Street, Taipei, 11031 Taiwan; 7 Taipei Medical University Research Center of Urology and Kidney, Taipei Medical University, 250 Wu-Hsing Street, Taipei, 11031 Taiwan; 8 Department of Urology, Shuang Ho Hospital, Taipei Medical University, 291 Zhongzheng Road, Zhonghe District, New Taipei City, 23561 Taiwan; 9 Institute of Biomedical Engineering and Nanomedicine, National Health Research Institute, Keyan Road, Zhunan, Miaoli City, 350 Taiwan; 10 Department of Chemical Engineering, National Taiwan University, 1 Roosevelt Road, Sec. 4, Taipei, 10617 Taiwan; 11 Department of Orthopedics and Trauma, Faculty of Medicine, University of Medicine and Pharmacy at Ho Chi Minh City, Ho Chi Minh City, Viet Nam; 12 International PhD Program in Biomedical Engineering, College of Biomedical Engineering, New Taipei City, Taiwan; 13 Cell Physiology and Molecular Image Research Center, Taipei Medical University-Wan Fang Hospital, 111 Hsing-Long Road, Sec. 3, Taipei, 11696 Taiwan; 14 Department of Chemical Engineering and Materials Science, Yuan Ze University, Chung-Li, Taoyuan, Taiwan; 15 Precision Medicine and Translational Cancer Research Center, Taipei Medical University Hospital, Taipei, 11031 Taiwan

**Keywords:** Phototherapy, Polypyrrole, Molybdenum disulfide, Rheumatoid arthritis, Immunomodulation

## Abstract

The prospective of percutaneous drug delivery (PDD) mechanisms to address the limitations of oral and injectable treatment for rheumatoid arthritis (RA) is increasing. These limitations encompass inadequate compliance among patients and acute gastrointestinal side effects. However, the skin’s intrinsic layer can frequently hinder the percutaneous dispersion of RA medications, thus mitigating the efficiency of drug delivery. To circumvent this constraint, we developed a strontium ranelate (SrR)-loaded alginate (ALG) phototherapeutic hydrogel to assess its effectiveness in combating RA. Our studies revealed that this SrR-loaded ALG hydrogel incorporating photoelectrically responsive molybdenum disulfide nanoflowers (MoS_2_ NFs) and photothermally responsive polypyrrole nanoparticles (Ppy NPs) to form ALG@SrR-MoS_2_ NFs-Ppy NPs demonstrated substantial mechanical strength, potentially enabling delivery of hydrophilic therapeutic agents into the skin and significantly impeding the progression of RA. Comprehensive biochemical, histological, behavioral, and radiographic analyses in an animal model of zymosan-induced RA demonstrated that the application of these phototherapeutic ALG@SrR-MoS_2_ NFs-Ppy NPs effectively reduced inflammation, increased the presence of heat shock proteins, regulatory cluster of differentiation M2 macrophages, and alleviated joint degeneration associated with RA. As demonstrated by our findings, treating RA and possibly other autoimmune disorders with this phototherapeutic hydrogel system offers a distinctive, highly compliant, and therapeutically efficient method.

**Supplementary Information:**

The online version contains supplementary material available at 10.1186/s12951-024-02877-8.

## Introduction

Rheumatoid arthritis (RA) is a chronic autoimmune disorder characterized by inflammation of the synovial joints, leading to pain, swelling, and progressive joint damage. This disorder affects about 1% of adults worldwide and can cause severe bone and cartilage loss, which eventually leads to disability [1]. Currently, corticosteroids and non-steroidal anti-inflammatory medications (NSAIDs), which are normally administered orally or by injections, are the mainstays of RA treatment [2]. The intended effects of these treatment modalities are to reduce inflammation and delay the course of RA. They accomplish this by focusing on processes associated with inflammation, such as lowering proinflammatory cytokines, impeding cell-mediated immunity, or suppressing expressions of genes linked to synovial collagenase [3, 4]. Despite advancements in treatment options, managing RA remains challenging due to limitations (injection-based complications and adverse acute gastrointestinal (GI) issues) associated with traditional oral and injectable therapies [5], which underscore the urgent need for alternative therapeutic approaches. Furthermore, the medications’ bioavailability may be notably decreased due to the harsh environment of the GI tract and the first-pass action of the liver [6].

Percutaneous drug delivery (PDD) mechanisms hold promise in overcoming these challenges by offering a mini-invasive and potentially more patient-friendly route of medication administration. Furthermore, PDD systems have garnered increasing attention as a potentially effective treatment for RA [7]. The PDD of medicines for RA gives the advantage of bypassing GI digestion associated with oral approaches and avoiding the discomfort of injections. Their popularity is further enhanced by their ease of self-administration. Small-molecule medications have shown promise in transdermal patches or gels, where they diffuse through the skin and provide anti-RA effects [8, 9]. Despite the potential advantages of PDD, the skin’s natural barrier presents a significant obstacle to efficient drug delivery, particularly for RA medications, thus demanding stringent requirements for the characteristics of pharmaceuticals employing this method. These include a log P-value between 1 and 3, a molecular weight of < 500 Da, and a specific balance of hydrogen bond donors and acceptors [7]. Additionally, to improve the effectiveness of medication administration through the skin, penetration enhancers and developments in nanoparticle (NP) technology have been used [10–12]. However, because of their often large molecular weights and strong hydrophilicity, the topical PDD of biological medicines in therapeutically meaningful quantities remains a difficulty [13].

In recent years, due to sufficient penetration depth, capacity to eradicate hyperplastic synovial tissues, and involvement in promoting the delivery of biomolecules, near-infrared (IR; NIR) light has garnered a lot of research interest [14–17]. It is well known that tissue regeneration can be aided by the mild localized heat produced by NIR radiation [16, 18]. Gold NPs [19], polydopamine [20], molybdenum disulfide (MoS_2_) [21], polypyrrole (Ppy) [22–36], carbon dots [37], and copper NPs [38] are among the photoresponsive biomaterials that have been successfully used as phototherapeutic agents, showing notable therapeutic outcomes in tumor inhibition, inflammation reduction, and tissue repair [39, 40]. Because of their simple synthesis, biocompatibility, high photothermal conversion efficiency, photoelectrical-induced immunomodulation, or capacity to combine medicinal drugs, MoS_2_ nanoflowers (NFs) [41, 42] and Ppy NPs stand out among these agents [29, 43]. It was observed that photothermally reactive Ppy activates cellular heat shock proteins (HSPs) [27], facilitating medication penetration and potentially mitigating the onset of arthritis by averting chondrocyte death. Furthermore, immunocytes can be regulated by photoelectrically responsive MoS_2_ [43], which may balance immunological responses and improve regeneration pathways in RA patients who are resistant to treatment.

Strontium ranelate (SrR) acupuncture was shown in previous research, including a number of clinical trials, to have potent anti-inflammatory and pain-relieving effects with minimal adverse effects for treating arthritis disorders [29]. Unfortunately, systemic or oral SrR administration can cause substantial GI adverse effects [44], which severely limits the drug’s therapeutic application for RA. These drawbacks might be overcome by transdermal distribution of SrR, such as by phototherapeutic methods. Research on the combined use of phototherapeutic methods with SrR administration appears to be rare as of yet. The use of the polysaccharide alginate (ALG) in conjunction with nanotherapies has emerged as a significant approach for creating biomimetic nanomedicines, which takes its cues from natural systems [45]. As a revolutionary platform, polysaccharide lading provides a simple top-down approach to building carriers with surface alterations that mimic intricate capabilities.

**Scheme 1:**
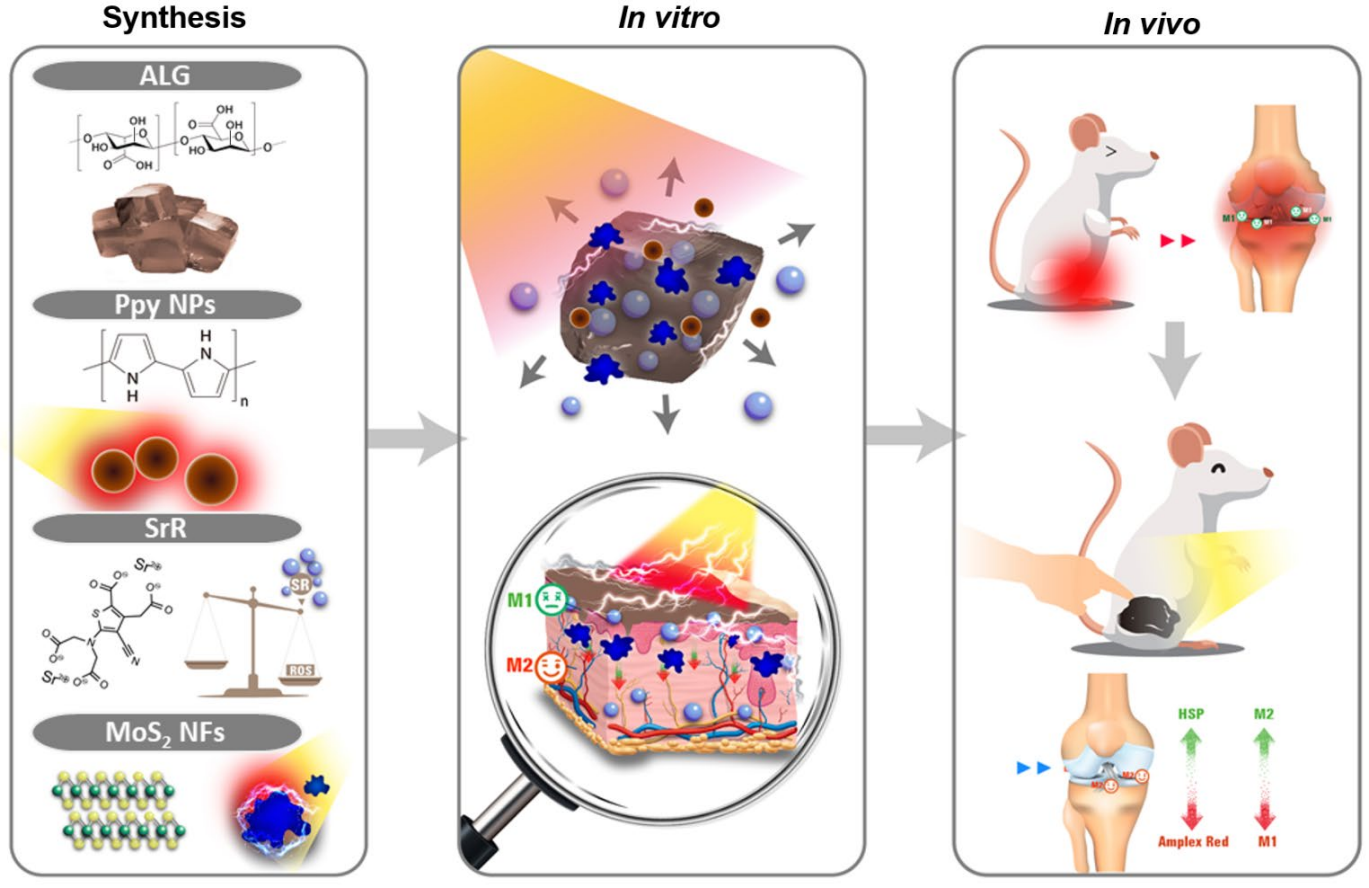
Schematic diagram demonstrating the innovative approach to synthesize composite biomaterials by integrating polypyrrole nanoparticles (Ppy NPs) and molybdenum disulfide nanoflowers (MoS_2_ NFs) into an alginate (ALG) hydrogel system to construct ALG@SrR-MoS_2_ NFs-Ppy NPs. The biomimetic combination of Ppy NPs and MoS_2_ NFs improved the mechanical strength of the gel by interacting in a coordinated manner and showed impressive photothermal and photoelectric conversion characteristics

In this current study, we hypothesized that phototherapeutic biomaterials in conjunction with PDD could improve the mitigation of RA development. In order to improve patient compliance and therapeutic efficacy, we set out to design a multimodal phototherapeutic hydrogel system for treating RA. Scheme 1 provides an illustration of this idea. SrR was incorporated into an ALG-based phototherapeutic hydrogel containing photoelectrical-responsive MoS_2_ NFs and photothermal-responsive Ppy NPs (ALG@SrR-MoS_2_ NFs-Ppy NPs), and the mechanical strength of the formulation was assessed through microencapsulation techniques. The effectiveness of this novel phototherapeutic hydrogel in treating RA was evaluated using a rodent model of zymosan-induced RA. As demonstrated by our research, ALG@SrR-MoS_2_ NFs-Ppy NPs guaranteed effective skin penetration of hydrophilic medication while also bolstering the hydrogel’s mechanical strength. Together, the phototherapeutic effects significantly slowed the course of RA by altering cellular immunity and lowering the release of proinflammatory cytokines. Furthermore, the hydrogel’s improved percutaneous penetration of this hydrophilic medication seemed to increase its therapeutic effect even further. Based on our investigation, phototherapeutic hydrogels for PDD could be a promising RA treatment strategy that improves patient compliance and therapy outcomes.

## Materials and methods

### Materials

ALG was CAS no. 9005-38-3. MoS_2_, pyrrole, ferric chloride, and polyethyleneimine (PEI) were from Merck (Taipei, Taiwan) and Sigma-Aldrich (St. Louis, MO, USA), L929 and RAW 264.7 cells were from ATCC (Manassas, VA, USA). Cell culture media and supplements were obtained from Gibco (Grand Island, NY, USA), including fetal bovine serum (FBS), alpha minimum essential medium (α-MEM), 3-(4,5-dimethylthiazol-2-yl)-2,5-diphenyltetrazolium bromide (MTT), and Dulbecco’s modified Eagle’s medium (DMEM). We procured additional biochemical reagents from multiple Taiwanese sources, such as Alfa Aesar, ThermoFisher Scientific, ECHO Chemical, and Scientific Biotech.

### Synthesis of Ppy NPs

Ppy NPs were synthesized using an established protocol [46]. Twenty milliliters of double-distilled water (ddH_2_O) was used to dilute cationic PEI (600 Da, 200 mg), and 6 M hydrochloric acid was used to lower the pH to 0.8. Ferric chloride hexahydrate (1 mL, 12.5 mg/mL) was gradually added to this solution after the addition of 12.5 µl of the pyrrole monomer. The polymerization process was allowed to proceed for 30 to 60 min. Using a 3500-Da MWCO dialysis membrane, the resultant black solution was purified by dialysis against ddH_2_O for 3 days, and it was oven-dried or lyophilized for further use.

### Synthesis of MoS2 NFs

MoS_2_ NFs in the crystal phase was synthesized using solvothermal techniques [47]. For MoS_2_, a solution of thiourea (0.0125 M) and molybdic acid (0.005 M) in deionized (DI) water (40 mL) was stirred until homogeneous. This mixture, along with a carbon fiber paper, was placed in a Teflon-lined hydrothermal autoclave reactor and heated to 180 °C for 24 h. The resulting MoS_2_ NFs were washed, dried, and used for further experiments.

### Preparation of ALG@SrR-MoS2 NFs-Ppy NPs

ALG was prepared to form a 670 mg solution by dissolving it in 6.7 mL of water at 50 °C. After 3 days of dialysis against DI water (MWCO: 3.5 kDa), the solution was filtered, centrifuged, and lyophilized at -80 °C. The resulting ALG powder was stored at -20 °C. To achieve homogeneity, ALG containing SrR, the produced MoS_2_ NFs, and Ppy NPs were combined to generate a composite mixture. The mixture was then agitated at 40 °C for 30 min. Ratios of ALG, SrR, prepared MoS_2_ NFs, and Ppy NPs in the ALG@SrR-MoS_2_ NF-Ppy NP formulation were adjusted to produce an optimal formulation that satisfied the required quality criteria.

### Characterization of ALG@SrR-MoS2 NFs-Ppy NPs

#### Hydrodynamic size, polydispersity index (PDI), and zeta potential

The hydrodynamic diameters and zeta potentials of MoS_2_ NFs and Ppy NPs were examined using a Zetasizer Nano ZS device (Malvern City, UK). A concentration of 0.1 mg/mL of NPs was distributed in Milli-Q water for the dynamic light scattering (DLS) investigation. After dispersing the NPs in 25 mM HEPES buffer at pH 7.2, a medically relevant pH level, the zeta potential was determined. Samples were vortexed and sonicated for 10 min in succession prior to measurements. After that, they were put into disposable cuvettes for the DLS analysis or disposable folded capillaries for zeta potential evaluations. The average hydrodynamic size, PDI, and zeta potential data—along with the standard deviation (SD) for each parameter—were provided for each of the three tests conducted on each sample.

#### Transmission electron microscopy (TEM)

A copper grid was coated with a 10-µL drop of a suspension containing MoS_2_ NFs, Ppy NPs, or ALG@SrR-MoS_2_ NFs-Ppy NPs in water to create TEM grids. This setup was left to dry overnight. A Hitachi HT-7700 Electron Microscope (Hitachi, Tokyo, Japan) fitted with a digital camera and operating at 80 kV was used for the imaging process. The successful fabrication, spherical shape, and size of the nanostructures were verified by the TEM examination. In particular, the size and surface potential analyses were performed at a regulated temperature of 22 °C using the Malvern Zetasizer Nano-ZS90 device.

#### Dynamic texture analysis (TA)

A dynamic TA (XT Plus, Godalming, UK) was used to determine the hydrogel’s room-temperature compression modulus. Samples were evaluated with different weight ratios of ALG, SrR, prepared MoS_2_ NFs, and Ppy NPs (of 95/0.18/0/1, 95/0.18/2/1, 95/0.18/4/1, 95/0.18/6/1, 95/0.18/8/1, and 95/0.18/10/1), each produced in 10-mm-diameter discs, in order to determine the optimal formulation of ALG@SrR-MoS_2_ NFs-Ppy NPs. The findings are displayed in terms of compressive stress in relation to the distance or working time. Furthermore, the rheological characteristics of the hydrogels were examined using a rheometer (AR2000ex, stress control, TA Instruments, New Castle, DE, USA) at temperatures ranging from 0 to 50 °C.

#### Thermogravimetric analysis (TGA)

A TGA was carried out utilizing the Netzsch DSC 404 F3 simultaneous TGA/DSC equipment (Netzsch 404 F3, Selb, Germany) in order to confirm surface alterations. Vacuum-dried samples were used, including ALG, ALG@SrR-Ppy NPs, and ALG@SrR-MoS_2_ NFs-Ppy NPs. milligrams of each sample was added to an alumina crucible for the analysis. Thermal data were obtained using argon as the carrier gas through a temperature range of 30 to 800 °C in an oxygen atmosphere (heating rate = 10 °K/min, flow rate = 30 mL/min).

#### X-ray powder diffraction (XRPD) measurements

X-ray diffractometer (XRD, Bruker AXS, Madison, WI, USA) was used for the XRPD studies. Cu-Kα radiation used in these investigations was adjusted to 45 kV and covered a 2θ range of 10° to 70°. Samples were evaluated spectroscopically using Fourier transform IR (FTIR) using a Nicolet iS 10 (Thermo Scientific, Waltham, MA, USA). With a resolution of 4 cm^− 1^, analyses were conducted in absorbance mode throughout a wavenumber spectrum of 4000 to 400 cm^− 1^.

#### Photothermal property analysis

The samples’ photothermal characteristics (ALG, MoS_2_ NFs, Ppy NPs, SrR, ALG@SrR-Ppy NPs, and ALG@SrR-MoS_2_ NFs-Ppy NPs) were assessed in 96-well plates. An Optoelectronics Tech Psu-iii device (Changchun City, China) was used to irradiate these materials with NIR light for 5 min at 808 nm and 2.0 W/cm^2^. Using an IR thermal camera (MET-FLTG300 + 2 NIR, S.E.A.T, Kaohsiung City, Taiwan), temperature variations and thermal pictures of the solutions during the irradiation process were captured and examined.

#### Photoelectric property analysis

An electric meter was used to evaluate the photoelectric characteristics of multiple test samples that were put on an indium tin oxide (ITO) substrate. An 808-nm NIR laser (Optoelectronics Tech PSU-III LED collimated at 808 nm) was used to repeatedly irradiate these samples, which included ALG, Ppy NPs, MoS_2_ NFs, SrR, ALG@SrR-Ppy NPs, and ALG@SrR-MoS_2_ NFs-Ppy NPs. As a control group, the ITO substrate itself was employed. Experimental data on the electrical current were collected over various time intervals.

#### Scanning electron microscopy (SEM)

An FE-SEM-Nova Nano 450 (Nova Nano, USA) with energy dispersive spectrometric (EDS) capability was used to take SEM images of samples after critical drying and gold coating.

#### In vitro drug release

To evaluate passive drug release, 200 mg of a hydrogel sample was placed into a 3500-Da dialysis bag and dialyzed against 1× phosphate-buffered saline (PBS; pH 7.4) with 10% bovine serum albumin (BSA). The surrounding fluid was periodically collected for analysis. SrR and ALG@SrR-MoS_2_ NFs-Ppy NPs (with a weight ratio of ALG/SrR/MoS_2_ NFs/Ppy NPs of 95/0.18/6/1) were submerged in 1× PBS (pH 7.4) enriched with 10% BSA and subjected to ultraviolet (UV) light (NIR light for 5 min at 808 nm and 2.0 W/cm²). Samples were then maintained at 37 °C, with the release of SrR tracked via optical spectroscopy at a wavelength of 321 nm. For comparison, a control was set up under identical conditions but without the exposure to NIR light.

### In vitro cell viability experiments

Mouse skin fibroblasts (L929, ATCC) or RAW 264.7 cells were used to assess the hydrogels’ cytotoxicity using MTT [32, 48] and live/dead cell staining assays in accordance with ISO standard 10,993. In 96-well plates, cells were grown in α-MEM supplemented with 10% FBS to evaluate the hydrogels’ biocompatibility. They were kept in an incubator with 5% CO_2_ at 37 °C, and medium was refreshed thrice weekly. A 96-well plate was seeded with 10^5^ cells/mL in each well. Following cell adhesion, hydrogel extracts were incubated in a 10:1 volume ratio of cell culture media to sample for 24 h at 37 °C and 5% CO_2_. After the extracts were filtered through a 0.22-µm filter, 100 µL of each extract was added to the wells and incubated for an additional 24 h. A microplate reader was used to measure the absorbance at 570 nm, and a control group of cells was grown without samples.

Cell viability was evaluated by following the manufacturer’s instructions using a Live/Dead Viability Kit (Invitrogen, Waltham, MA, USA). After seeding and cultivating the cells (10^5^), 0.2 mL of the test solution was added to each sample, and samples were incubated for 45 min at room temperature. Ethidium homodimer (EthD)-1, which stains dead cells red, and calcein-AM, which stains living cells green, were both present in the assay fluid. Following incubation, cells were examined under a Leica fluorescent microscope (Germany) and cleaned with PBS. The ImageJ program (National Institutes of Health, Bethesda, MD, USA) was used to quantify living and dead cells. Furthermore, standard immunofluorescence (IF) methods using fluorescently conjugated anti-HSP primary antibodies were used to quantify HSP expressions in control and experimental group cells. Images were taken using a Leica fluorescence microscope.

### Macrophage polarization and biochemical experiments

RAW 264.7 cells were cultured in DMEM supplemented with 10% FBS and maintained at 37 °C in an incubator with 5% CO_2_. These cells were used to assess the anti-inflammatory effects and cell polarization. Using fluorescent methods, the polarization behavior of macrophages (RAW 264.7 cells) in response to the implanted microenvironment was examined. On coverslip surfaces, 10^5^ cells/well were used for seeding. After they were confluent and had adhered, they were stimulated for 12 h with lipopolysaccharide (LPS, 1 µg/mL) to become the M1 type. Cells that had been activated with LPS were next subjected to a hydrogel extraction medium for 1 day. After that, cells were rinsed and treated for 30 min with fluorescent-tagged cluster of differentiation 206 (CD206; an M2 marker) and CD86 (an M1 marker). Leica fluorescence microscopes were used to record the fluorescence results. Using ImageJ software, the M1 and M2 fluorescence intensities in cells were measured. An Amplex red probe from the ROS kit (ADHP, Beyotime, Shanghai, China) was used for intracellular reactive oxygen species (ROS) measurements. After being treated with the hydrogel, cells were viewed under a Leica fluorescence microscope and subsequently incubated with Amplex red and DAPI. Using ImageJ software, the intensity of cellular ROS fluorescence was measured.

### In vivo investigations

#### Establishment of an arthritis model and administration of formulations

Guidelines authorized by Taipei Medical University (TMU)’s Institutional Animal Care and Use Committee (IACUC) were followed for conducting experiments on animals. A zymosan-induced arthritis mice model was established utilizing an existing protocol [49]. Male mice weighing roughly 20 ~ 25 g were purchased from BioLASCO (Taipei, Taiwan). Every 3 days, mice under anesthesia with 1–4% isoflurane inhalant had 100 µL of zymosan injected intra-articularly into their knee joints. The zymosan concentration was 15 mg/mL in PBS.

#### Biodistribution evaluations

To investigate the in vivo permeation and biodistribution, FITC and cyanine 5 (Cy5) were employed as models for hydrophilic low-molecular-weight drugs. Formulations for in vivo tracking included these fluorescent markers. Following administration of these fluorescent formulations to ICR mice with RA, an in vivo imaging system (IVIS) was used to track biodistributions in the mice at predetermined intervals. Topical administration comprised putting 30 mg of different samples (ALG@SrR-Ppy NPs with FITC, SrR + fluorescein isothiocyanate (FITC), and ALG@SrR-MoS_2_ NFs-Ppy NPs with FITC) in the vicinity of the knee joint and taping the area to keep the sample in place. NIR treatment continued for 5 min after this.

ALG@SrR-MoS_2_ NFs-Ppy NPs containing Cy5 + NIR were applied transdermally to the knee joint of an RA animal in order to measure the skin penetration (5 min). Mice were killed 2 h after treatment, and skin samples were taken from the application areas for a histological examination. To determine the distribution of fluorescence within the layers of skin, samples were viewed under a fluorescence microscope. Furthermore, skin tissues were stained for 30 min at 4 °C in the dark with a macrophage marker (F4/80, Biolegend). After that, samples were photographed under a Leica fluorescence microscope.

#### Radiographic, biochemical, and histological analyses

Topical administration was done by applying 30 mg of different samples (ALG@SrR-Ppy NPs, ALG@SrR-MoS_2_ NFs-Ppy NPs, and SrR) in the vicinity of the knee joint, then taping the area to keep the sample in place. NIR treatment was repeated for 5 min each day. Gross knee photography, and histological, radiographic, and magnetic resonance imaging (MRI) investigations, in addition to behavioral evaluations, were used to monitor the development of arthritis in this animal model. The in vivo photothermal response of ALG@SrR-MoS_2_ NFs-Ppy NPs to NIR treatment was assessed using a thermal imaging camera.

PharmaScan 7.0 T system (BioSpin MRI, Bruker, Germany) was used to perform MRI scans. Isoflurane (1–4%) was used to induce anesthesia in arthritic mice, whose body temperature was maintained at 36–37 °C. MRI parameters were as follows: 16 slices, each 1.0 mm thick; a 40-mm coil; and a 40 × 40-mm field of view. Knee tissues of CO_2_-sacrificed mice were removed for paraffin sectioning in order to conduct histological investigations. Sections were treated with hematoxylin and eosin (H&E), IF, or immunohistochemical (IHC), Alcian blue (AB, Sigma, St. Louis, MO, USA) staining for acid proteoglycans in the knee cartilage, M1 (CD86, 1:100, Abcam), M2 (CD206, 1:100, Abcam), T cell antibodies (CD8+, 1:100, Abcam), T cell antibodies (CD3+, 1:100, Abcam), interleukin (IL)-6 antibodies (Abcam), and HSP antibodies (HSP70, 3:1000, Abcam). ROS levels were determined using Amplex red. The heart, liver, lungs, kidneys, and spleen were also collected, sectioned, and subjected to H&E staining. Each experiment involved three mice per group.

### Statistical analysis

The study’s quantitative data are presented as the average ± standard deviation (SD) of a minimum of three replicate experiments (*n* ≥ 3). We used the two-way analysis of variance (ANOVA) or the Student’s t-test to determine the statistical significance among several groups. GraphPad Prism software vers. 5.04 for Windows was used to do this analysis (Dotmatics, Boston, MA, USA). Thresholds of statistical significance were set to *p* < 0.05 (*), *p* < 0.01 (**), *p* < 0.001 (***), and *p* < 0.0001 (****).

## Results and discussion

### Preparations and characterizations of ALG@SrR-MoS2 NFs-Ppy NPs

**Fig. 1:**
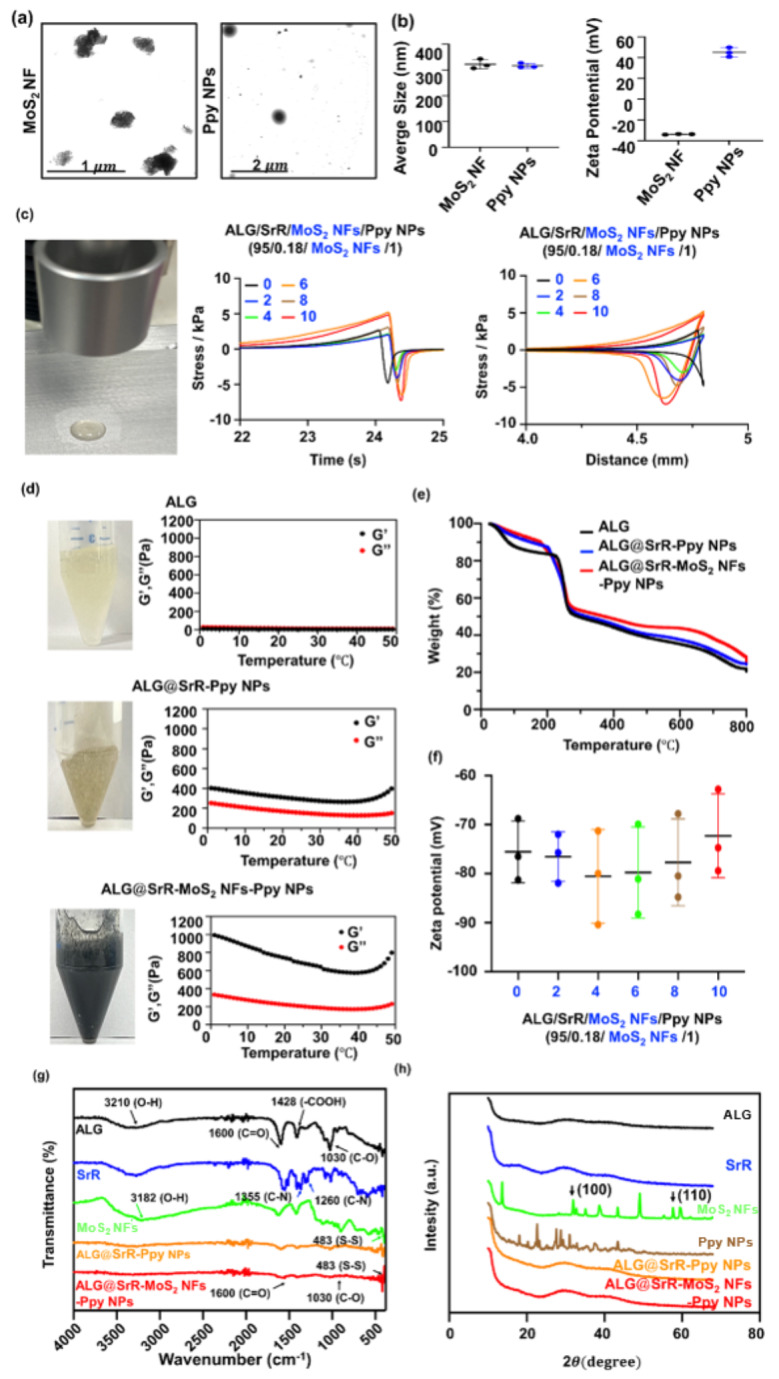
A comprehensive overview of the fabrication and characterization of strontium ranelate incorporated into alginate-based hydrogels containing molybdenum disulfide nanoflowers and polypyrrole nanoparticles (ALG@SrR-MoS_2_ NFs-Ppy NPs). (**a**) Transmission electron microscopic (TEM) images provided detailed visualization of the morphologies of MoS_2_ NFs and Ppy NPs. (**b**) Dynamic light scattering (DLS) tests were performed to evaluate the hydrodynamic sizes of MoS_2_ NFs and Ppy NPs. (**c**) A texture analysis (TA) was conducted to examine the mechanical properties of formulations comprising ALG, SrR, MoS_2_ NF, and Ppy NPs in specific ratios (95/0.18/0/1, 95/0.18/2/1, 95/0.18/4/1, 95/0.18/6/1, 95/0.18/8/1, and 95/0.18/10/1). (**d**) Rheological experiments were carried out to assess the viscoelastic properties of ALG, ALG@SrR-Ppy NPs, and ALG@SrR-MoS_2_ NFs-Ppy NPs. (**e**) Thermogravimetric analysis (TGA) tests determined the thermal stability and decomposition characteristics of ALG@SrR-MoS_2_ NFs-Ppy NPs. (**f**) Zeta potential measurements were performed to understand the surface charge properties of the formulations with varying ratios of ALG, SrR, MoS_2_ NFs, Ppy NPs. (**g**) A Fourier transform infrared (FTIR) spectroscopic analysis provided insights into the chemical structures of and bonding among ALG, SrR, MoS_2_ NFs, ALG@SrR-Ppy NPs, and ALG@SrR-MoS_2_ NFs-Ppy NPs. (**h**) X-Ray diffraction (XRD) assessments offered information on the crystalline or amorphous nature of ALG, SrR, MoS_2_ NFs, Ppy NPs, ALG@SrR-Ppy NPs, and ALG@SrR-MoS_2_ NFs-Ppy NPs. These analyses collectively contributed to a deeper understanding of the physical, chemical, and thermal properties of the ALG@SrR-MoS_2_ NF-Ppy NP hydrogel, paving the way for potential biomedical applications

MoS_2_ NFs and Ppy NPs are exceptional phototherapeutic nanomaterials widely used in regenerative medicine and drug-delivery systems [50–52]. The synthesized MoS_2_ NFs and Ppy NPs were characterized using TEM to examine their morphology and size. As depicted in Fig. 1a, MoS_2_ NFs exhibited a flower-like structure with a consistent size distribution of around 220 nm. Conversely, Ppy NPs showed a spherical shape, averaging 210 nm in diameter. DLS measurements (Fig. 1b) revealed the hydrodynamic sizes of these NPs in DI water: approximately 320 nm for anionic MoS_2_ NFs (ca. -30 mV) and 317 nm for cationic Ppy NPs (ca. +40 mV), slightly larger than the sizes observed in TEM images. Results from DLS measurements often indicate a larger hydrated particle size for NPs compared to the sizes observed using TEM. This discrepancy typically arises because DLS measures the hydrodynamic diameter, which includes any water molecules bound to the particle surface, whereas TEM provides a direct visual measurement of the dry particle, free of any surrounding hydration layer [53].

To enhance their functionality, a hydrogel containing Ppy NPs and MoS_2_ NFs along with SrR (ALG@SrR-MoS_2_ NFs-Ppy NPs) was developed using an ultrasonic fabrication method, as shown in Fig. 1c-h for further investigations. In the formulation experiment, we meticulously designed the composition of ALG, SrR, MoS_2_ NFs, and Ppy NPs in specific mass ratios (of 95/0.18/0/1, 95/0.18/2/1, 95/0.18/4/1, 95/0.18/6/1, 95/0.18/8/1, and 95/0.18/10/1). In the case of static coordination polymers, metal composites generally exhibit metallic bonding [54]. When metals form complexes with ligands, such as in coordination polymers or metal-organic frameworks, they can exhibit strong coordinate bonding due to the metal’s ability to accept electron pairs from ligands. For the formulation tests, different mass ratios of MoS_2_ nanofibers were evaluated while maintaining the composition of ALG, SrR, and Ppy NPs constant at 95/0.18/1.

The resulting hydrogel samples underwent a texture analysis (TA) to evaluate their mechanical properties (Fig. 1c). Outcomes from the TA provided insightful data, revealing an increasing tendency in anti-compressive strength and anti-adhesive properties of the gel as the amount of MoS_2_ NFs increased. Given the minimal differences observed in the TA experiment (ca. 5 kPa of anti-compressive/adhesive strength) between formulations with ratios of 95/0.18/6/1 and 95/0.18/10/1, and to optimize material usage, the formulation with the 95/0.18/6/1 ratio was selected for subsequent use (ALG@SrR-MoS_2_ NFs-Ppy NPs). Rheological assessments conducted on the ALG@SrR-MoS_2_ NF-Ppy NP hydrogel further substantiated its mechanical properties. As depicted in Fig. 1d, rheological data revealed that the ALG@SrR-MoS_2_ NF-Ppy NP formulation exhibited an elevated storage modulus (G’) in the range of approximately 800 to 1000 Pa, and a loss modulus (G”) of 200 to 400 Pa. These values were notably higher compared to those of the ALG and ALG@SrR-Ppy NP groups. Furthermore, the dominance of G’ over G” within the ALG@SrR-MoS_2_ NF-Ppy NP system signified a predominantly elastic nature of the hydrogel. This characteristic indicated the formation of a gel system with enhanced elasticity, a crucial parameter for various practical applications.

The TGA is an instrumental technique utilized to determine thermal degradation temperatures of materials and to verify the presence of distinct polymer components within samples. Thermograms in Fig. 1e illustrate mass loss as a function of temperature for the ALG, ALG@SrR-Ppy NP, and ALG@SrR-MoS_2_ NF-Ppy NP hydrogels. Consistent with previous findings, the initial mass loss observed near 100 °C was attributed to the evaporation of water molecules associated with hydrophilic polymer chains [55, 56]. TGA results (Fig. 1e) at temperatures of 400 to 800 °C demonstrated enhanced thermal stability in the ALG@SrR-MoS_2_ NF-Ppy NP polymeric hydrogel compared to the ALG and ALG@SrR-Ppy NP counterparts. Notably, the ALG@SrR-MoS_2_ NF-Ppy NP hydrogel exhibited superior thermal resistance, likely due to the synergistic cross-linking effect imparted by the MoS_2_ NF component within the gel matrix. This observation suggested that the incorporation of MoS_2_ NFs significantly contributed to the thermal robustness of the hydrogel, a critical factor for its application in environments with varying thermal conditions. Correlations of the TA, rheological, and TGA data underscore the significant impact of MoS_2_ NFs in enhancing the structural integrity and functional characteristics of these ALG polysaccharide hydrogel formulations. Utilization of metal-based coordination bonds as dynamic cross-linkers in the fabrication of self-healing hydrogels represents a significant advancement in materials science [57, 58]. These bonds impart notable properties to hydrogels, such as enhanced mechanical strength and the ability to self-repair, making them highly relevant for a variety of applications. Exploration of metal coordination in hydrogel formation provides crucial insights and opens up new avenues for innovation and development in the field of smart materials. The ALG@SrR-MoS2 NF-Ppy NP hydrogel formulation significantly improved the mechanical, anti-compression, and adhesive characteristics of the hydrogels. This enhancement boosted stability, facilitated prolonged drug release, and ensured sustained application to the targeted area. Additionally, the reinforced mechanical properties of the hydrogels enhanced drug penetration, fostering improved cellular interactions [59]. Clinical trials demonstrated the effectiveness of this hydrogel therapy, with results showing complete therapeutic success [60].

Zeta potential (Fig. 1f) measurements for the ALG@SrR-Ppy NP and ALG@SrR-MoS_2_ NF-Ppy NP formulations revealed a negative charge characteristic, which was attributed to the presence of ALG. This observation aligns with findings previously reported in the literature [61, 62]. The presence of ALG, known for its anionic nature, significantly contributed to the overall charge profile of these composite materials, influencing their interactions with biological systems and their potential applications in various fields [63].

FTIR analysis of the ALG@SrR-MoS_2_ NF-Ppy NP formulation revealed distinct characteristic peaks, indicative of the composite material’s composition (Fig. 1g). Spectra show characteristic O-H stretching of ALG at 3210 cm^− 1^; characteristic C-O bending at 1030 cm^− 1^ which was present in the individual components of ALG, SrR, and MoS_2_; and C-N bending at 1355 cm^− 1^, S-  S stretching at 483 cm^− 1^, C=O stretching at 1600 cm^− 1,^ and COOH stretching at 1428 cm^− 1^ which were all present in individual products. The hydrogel nerk may have formed new chemical bonds, or existing bonds may have been rearranged as indicated by the absence or shifting of peaks linked to its initial ingredients. These spectral features confirmed the successful integration of individual components into the composite hydrogel, providing a molecular-level understanding of its structure. The ALG@SrR-Ppy NP group exhibited characteristic FTIR peaks including C = O stretching at 1600 cm^− 1^ and C-O stretching at 1030 cm^− 1^, attributable to the ALG component, and C-N stretching observed at 1355 and 1260 cm^− 1^, associated with SrR. For the ALG@SrR-MoS_2_ NF-Ppy NP group, similar peaks were noted for ALG and SrR. Additionally, this group featured an S-S stretching peak at 483 cm^− 1^, indicative of the presence of MoS_2_ NFs. These spectral signatures confirmed the successful incorporation of each component within the composite hydrogels, elucidating the distinct molecular interactions that contributed to their structural integrity and functionality.

In the XRD analysis (Fig. 1h), distinct peaks observed at approximately 33° and 57° were respectively assigned to the (1 0 0) and (1 1 0) crystallographic planes of MoS_2_ [64]. These peaks are indicative of the crystalline structure of MoS_2_ within the sample. Additionally, XRD patterns of Ppy NPs exhibited sharp diffraction peaks, denoting a highly ordered molecular arrangement within the films, which is a hallmark of their high crystalline quality. Conversely, XRD spectra for ALG@SrR-MoS_2_ NFs-Ppy NPs predominantly exhibited characteristics of amorphous materials. The lack of sharp peaks in these spectra suggested that these compounds and the major composites containing amorphous ALG did not possess a long-range crystalline order, which is typical of amorphous substances.

Moreover, the TEM analysis as illustrated in Fig. S1a provides additional verification that the ALG@SrR-MoS_2_ NF-Ppy NP composition included polymeric filaments. These filaments effectively encapsulated the MoS_2_ NFs and Ppy NPs, highlighting the structural integration of the components within the hydrogel matrix. This encapsulation supported the stability and functionality of the composite material.

**Fig. 2:**
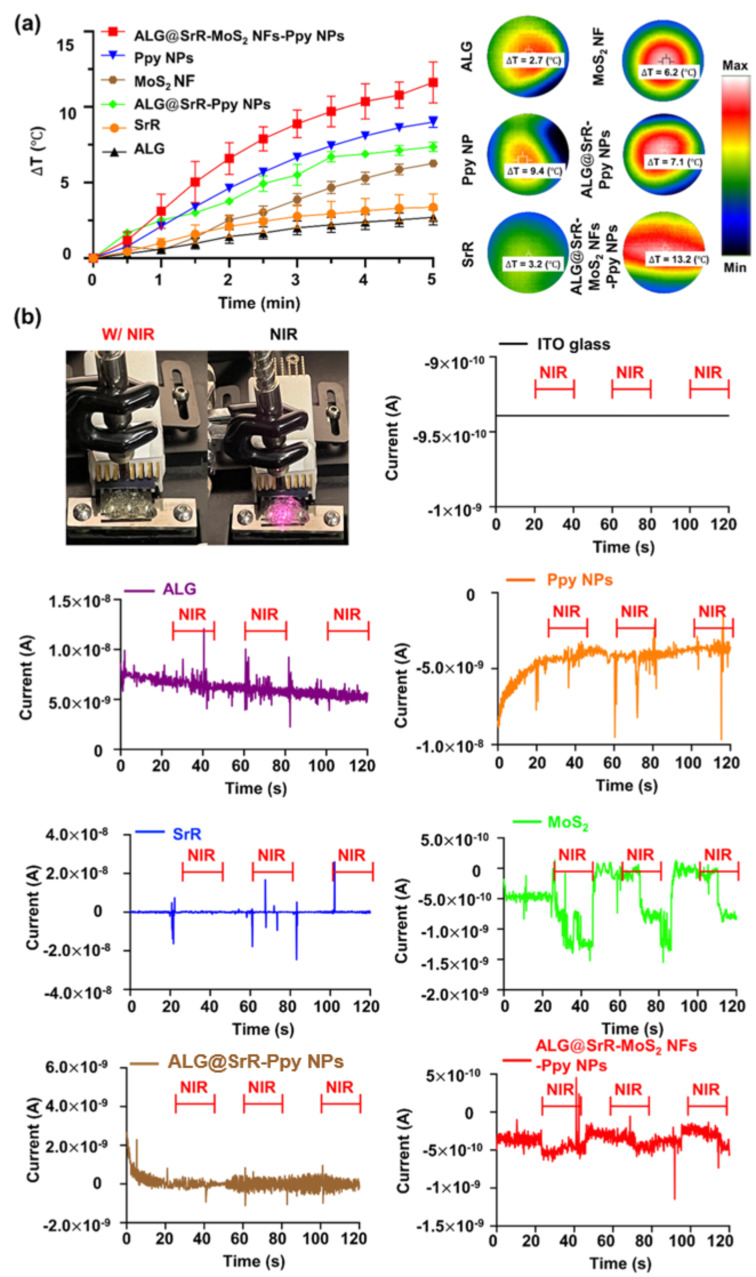
Findings of the photoresponsivity, encompassing both photothermal and photoelectric responses of various materials. (**a**) Photothermal results illustrate the thermal behavior of alginate (ALG), strontium ranelate (SrR), molybdenum disulfide nanoflowers (MoS_2_ NFs), polypyrrole nanoparticles (Ppy NPs), ALG@strontium ranelate (SrR)-Ppy NPs and the ALG@strontium ranelate (SrR)-MoS_2_ NF-Ppy NP composite. These were assessed under repeated near-infrared (NIR) irradiation (808 nm, 1.0 W/cm²) to evaluate their heat-generation capabilities. (**b**) Photoelectric results demonstrated the electrical responses of the same materials under similar conditions of frequent NIR irradiation. This part of the study focused on examining changes in electrical conductivity and the photoelectric efficiency during on-off cycles of NIR exposure. Together, these results offer comprehensive insights into the photothermal and photoelectric properties of ALG@SrR-MoS_2_ NFs-Ppy NPs, showcasing their potential applications in fields where photoresponsive behavior is crucial. Some study’s quantitative data are presented as the average ± standard deviation (SD) of a minimum of three replicate experiments (*n* ≥ 3)

In a detailed analysis of the photothermal properties, heating profiles and thermal images (Fig. 2a) were obtained under 808-nm laser irradiation of various samples. This study revealed a time-dependent temperature increase in MoS_2_ NFs, Ppy NPs, and ALG@SrR-MoS_2_ NFs-Ppy NPs compared to SrR and ALG. Upon exposure to NIR irradiation, both ALG and SrR exhibited a minimal temperature increase of about 3 °C. In contrast, MoS_2_ NFs showed a slightly higher temperature rise of approximately 6 °C under the same conditions. Specifically, temperatures of Ppy NP and ALG@SrR-Ppy NP samples rose by approximately 8 °C within 5 min, situating them within the mild-hyperthermia therapeutic window, which is known to be non-detrimental to normal tissues [65, 66]. The temperature of the ALG@SrR-MoS_2_ NF-Ppy NPs rose by more by 13.2 °C as shown by thermal camera images. These findings highlight the capability of MoS_2_ NFs in augmenting the photothermal efficiency of Ppy NPs within the ALG@SrR-MoS_2_ NF-Ppy NP composite, confirming prior research on their effective light-harvesting properties [67–69].

Further investigations were conducted into the photoelectric performance of these samples, when coated onto ITO substrates (Fig. 2b). The 808-nm NIR laser served as the optical source, with a primary focus on maximizing the photoresponse. Current-time curves presented in Fig. 2b compare the photoelectric behaviors of samples’ on-off NIR excitation. Results displayed in Fig. 2b demonstrate the exceptional photoelectric stability of MoS_2_ NFs and ALG@SrR-MoS_2_ NFs-Ppy NPs, as evidenced by consistent current variations across multiple irradiation cycles. In contrast, the ITO-coated ALG, Ppy NP, and SrR groups exhibited minimal current fluctuations during NIR irradiation cycles, aligning with findings from prior research on the photoelectric effects of MoS_2_ composites [70–72]. Overall, our findings highlight the high photothermal and photoelectric conversion efficiencies of ALG@SrR-MoS_2_ NFs-Ppy NPs, as substantiated by both thermal and electrical measurements.

The ALG@SrR-MoS_2_ NF-Ppy NP composite exhibited improved NIR photothermal and photoelectrical characteristics. Through NIR-photothermal activation, this composite can enhance drug delivery deep into tissue lesions, attract immune cells like macrophages, and stimulate cellular expressions of HSPs which contribute to tissue regeneration. Additionally, its NIR-photoelectric properties could aid in polarizing macrophages towards an anti-inflammatory M2 phenotype, potentially mitigating the progression of RA.

The release kinetics of SrR from ALG@SrR-MoS_2_ NF-Ppy NPs was next analyzed upon photo-irradiation. According to the drug release experimental data (Fig. S1b), free-form SrR was quickly released from the dialysis bag. After photo-irradiation, a notable increase in the absorbance intensity at a wavelength of 321 nm was observed, reaching a saturation point within 24 h as shown in Fig. S1b. By adjusting the photo-irradiation duration, release kinetics of SrR from ALG@SrR-MoS_2_ NF-Ppy NPs were optimized, achieving maximum drug release as documented in (Fig. S1b). Specifically, ca. 70% cumulative release of SrR was achieved after just 10 min of NIR exposure (Fig. S1b). Conversely, without NIR exposure, there was minimal release of SrR from ALG@SrR-MoS_2_ NF-Ppy NPs. This method of controlled and triggered drug release, which is modulated through photo-irradiation, allows for a wide range of customizable drug release profiles that can be finely adjusted over time.

Light serves as an effective external stimulus for drug release, offering precise spatial and temporal control. Compared to other remote triggers used in drug delivery, light is less invasive and requires no complex equipment, and drug release can be easily adjusted by altering the light’s wavelength, power density, and exposure duration. The ALG@SrR-MoS_2_ NFs-Ppy NPs demonstrated tunable drug release kinetics between ‘on’ (light exposure) and ‘off’ (no light exposure) states. However, the absence of photo-irradiation may lead to unintended drug leakage from the polymer reservoir through passive diffusion, raising concerns about the system’s long-term stability and clinical relevance.

In the design of the phototherapeutic hydrogel system used in this study, MoS_2_ NFs and Ppy NPs were integrated due to their distinct and complementary phototherapeutic properties, enhancing the system’s overall functionality. MoS_2_ NFs are known for their excellent photoelectric conversion efficiency, allowing them to effectively absorb and convert NIR light into heat, which can facilitate drug delivery by enhancing tissue permeability. On the other hand, Ppy NPs were included for their strong photothermal properties, which can influence cellular behavior at the molecular level, potentially favorably modulating immune responses in the context of inflammatory diseases like RA. Combining these types of NPs not only leveraged their individual strengths but also created a synergistic effect that enhanced the hydrogel’s capability to efficiently deliver drugs through the skin. The incorporation of both photothermal and photoelectric materials allowed for a multifaceted approach to therapy—photothermal effects help in the deeper penetration of drugs, while photoelectric effects contribute to local modulation of the immune environment.

The mechanism by which the phototherapeutic hydrogel promotes percutaneous delivery of SrR involves several key interactions and reactions facilitated by the properties of the hydrogel components under NIR irradiation. When exposed to NIR light, Ppy NPs generate mild heat, which can temporarily disrupt the skin barrier, reducing its resistance and allowing larger hydrophilic molecules like SrR to penetrate into deeper skin layers. Simultaneously, the photoelectric effects generated by MoS_2_ NFs under the same NIR exposure can potentially alter local cell functions, enhancing drug uptake. Experimental data suggested that NIR activation of the hydrogel not only promoted increased SrR delivery through the skin but might also have affected the drug’s stability and release kinetics. It is crucial to evaluate whether these interactions alter the chemical properties of SrR once it is released from the hydrogel. Studies involving in vitro and in vivo degradation profiles, along with pharmacokinetic analyses, are essential to understanding how the photothermal and photoelectric properties impact SrR once it is percutaneously administered.

Furthermore, controlled drug release experiments under various conditions of NIR irradiation can provide deeper insights into the tunable release capabilities of the hydrogel. By systematically varying the wavelength, intensity, and duration of NIR exposure, researchers can map out precise drug release profiles, optimizing therapeutic outcomes while minimizing potential side effects. This approach ensures that the hydrogel system can be adapted for personalized therapy, meeting specific needs of individual RA patients.

**Fig. 3:**
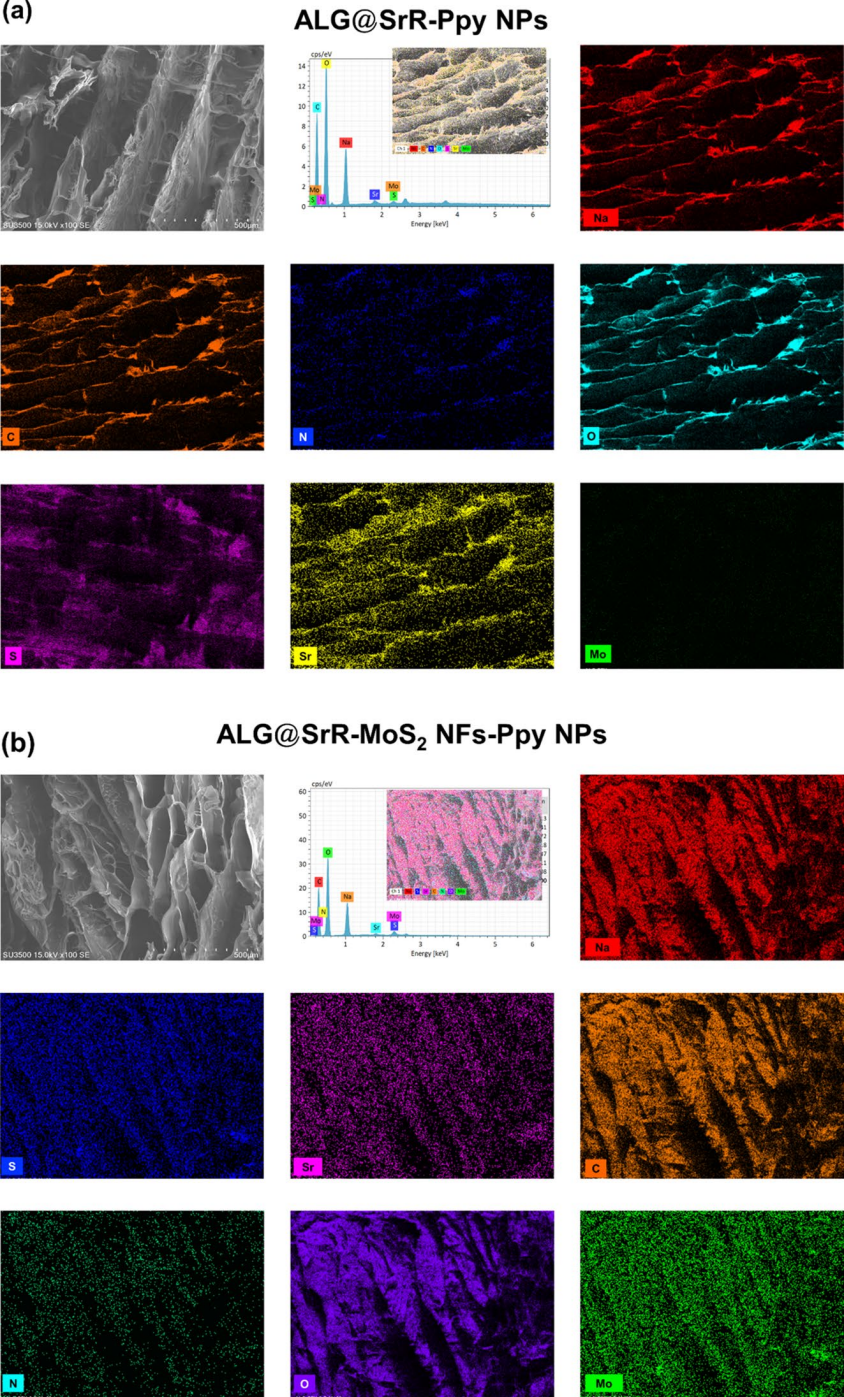
SEM-EDS analysis of (**a**) alginate-incorporated strontium ranelate-polypyrrole nanoparticle (ALG@SrR-Ppy NPs) and (**b**) ALG@SrR-molybdenum disulfide nanoflower (MoS_2_ NFs)-Ppy NPs hydrogels. High-resolution SEM images illustrating microstructural differences between ALG@SrR-Ppy NPs and ALG@SrR-MoS_2_ NFs-Ppy NPs, with the latter displaying a regular and distinct porous morphology. EDS spectra highlighting the elemental composition of the hydrogels, with ALG@SrR-Ppy NPs showing a predominance of oxygen (O), carbon (C), nitrogen (N), and strontium (Sr). ALG@SrR-MoS_2_ NFs-Ppy NPs revealed similar elemental contents with the addition of sulfur (S) and molybdenum (Mo), indicating the successful incorporation of MoS_2_ NFs into the hydrogel nerk. The figure demonstrates the homogeneity and distinct structures within the hydrogel composites, validating their potential application in targeted drug delivery and regenerative medicine

The current study meticulously examined the microstructural characteristics and elemental compositions of ALG@SrR-Ppy NP and ALG@SrR-MoS_2_ NF-Ppy NP samples using SEM coupled with EDS. To optimize the clarity of SEM imaging and the precision of EDS quantification, samples were subjected to a preparatory coating process, the details of which are shown in Fig. 3. SEM-EDS data revealed that the ALG@SrR-MoS_2_ NFs-Ppy NPs (Fig. 3b) exhibited a well-defined, regular porous structure, offering a contrast to the morphology of the ALG@SrR-Ppy NP group (Fig. 3a). The further detailed quantitative elements within the ALG@SrR-Ppy NPs, identifying oxygen (O) as the major constituent at 54.5%, followed by carbon (C) at 35.2%, nitrogen (N) at 1.9%, and strontium (Sr) at 0.7%. The composition of the ALG@SrR-MoS_2_ NFs-Ppy NPs was similar but also included sulfur (S) at 0.7% and molybdenum (Mo) at 0.5%, with a slightly diminished presence of strontium (Sr) at 0.6%. This elemental analysis confirmed the integration of the nanomaterials within the hydrogel matrix and underscored the consistency of the compositional framework between the different hydrogel formulations.

### In vitro assessments of the biosafety and therapeutic efficacies of ALG@SrR-MoS2 NFs-Ppy NPs

**Fig. 4:**
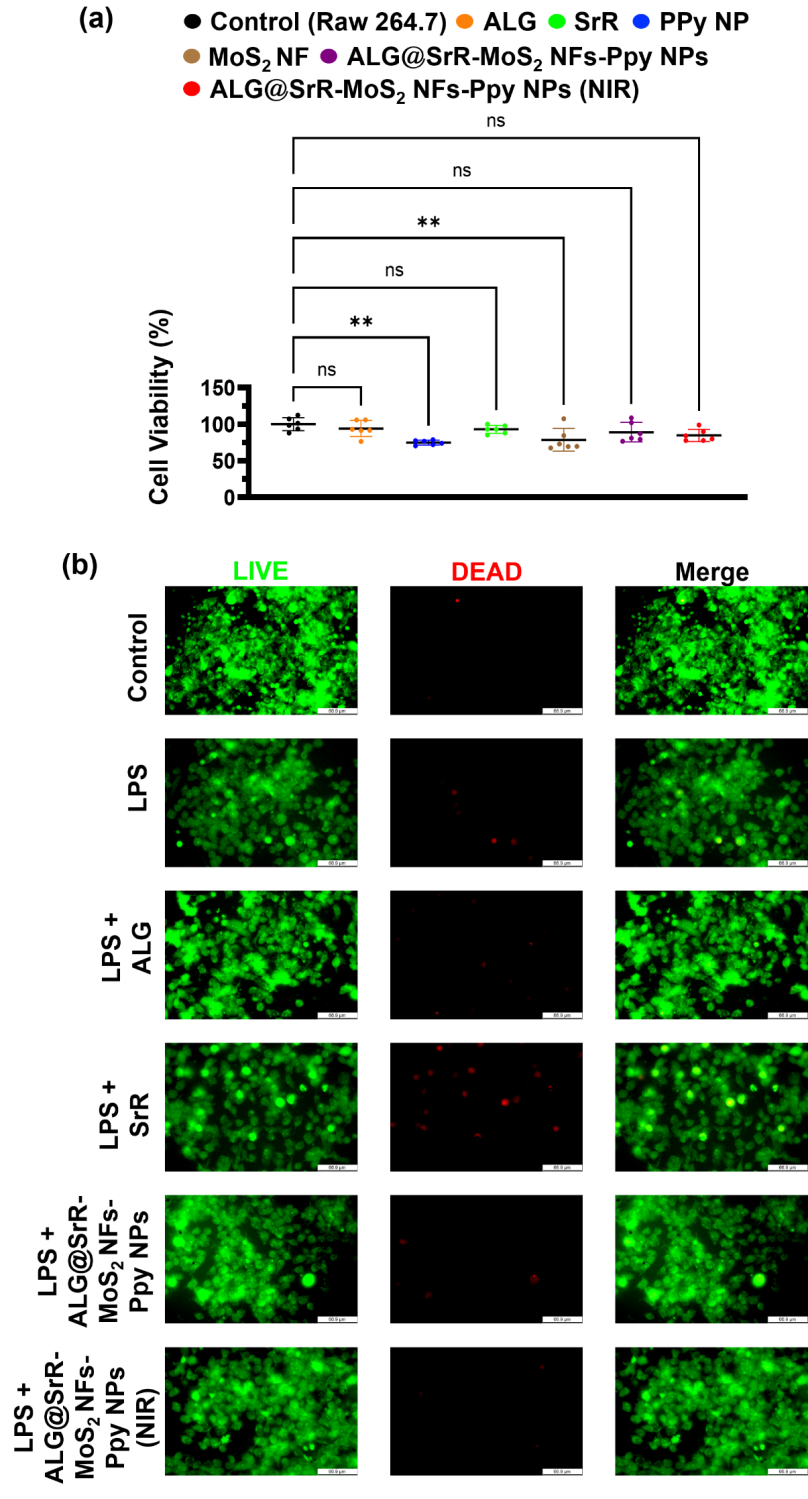
In vitro investigation of the cellular viability and apoptosis in RAW 264.7 cells. (**a**) MTT assay results revealed the biocompatibility of the samples, indicating minimal cytotoxicity across all tested formulations including alginate (ALG), polypyrrole (Ppy) nanoparticles (NPs), molybdenum disulfide (MoS_2_) nanoflowers (NFs), strontium ranelate (SrR), and ALG@SrR-MoS_2_ NFs-Ppy NPs, both with and without near-infrared (NIR) irradiation. ALG/SrR/MoS_2_ NFs/Ppy NPs had a weight ratio of 95/0.18/6/1. (**b**) Fluorescence microscopic images demonstrating apoptosis using a calcein-AM/ethidium homodimer assay. Live cells are indicated by green fluorescence, while apoptotic cells are marked by red fluorescence. The group treated with ALG@SrR-MoS_2_ NFs-Ppy NPs exhibited a slight increase in red fluorescence, suggesting marginal apoptosis with the majority of cells remaining viable. Statistical significance thresholds were established at *p* values of < 0.05 (*), < 0.01 (**), and < 0.001 (***)

Mitochondrial activity is intricately linked to cell apoptosis. The biocompatibility of the various samples, including ALG, Ppy NPs, MoS_2_ NFs, SrR, ALG@SrR-MoS_2_ NFs-Ppy NPs, and their counterparts subjected to NIR irradiation, was evaluated using MTT assays. All tested samples exhibited negligible toxicity towards RAW 264.7 cells, as shown in Fig. 4a. The roles of individual components in cell viability were analyzed, and cells were viable in the presence of every individual component. Even under NIR stimulation, the ALG@SrR-MoS_2_ NFs-Ppy NPs were not cytotoxic. Results demonstrated that more than ca. 75% of cells were viable after treatment, which proves the developed NFs were cytocompatible.

In this study, a calcein-AM/EthD-1 assay was employed to evaluate apoptosis among cultured cells. Apoptotic cells were marked by red fluorescence emitted from EthD-1 staining, whereas live cells fluoresced green due to calcein-AM. The control set, consisting of untreated RAW 264.7 cells, displayed prominent green fluorescence, indicating minimal apoptosis as shown in Fig. 4b. Different experimental conditions were tested, including RAW 264.7 cells treated with LPS, LPS combined with ALG, LPS with SrR, LPS with ALG@SrR-MoS_2_ NFs-Ppy NPs, and LPS with ALG@SrR-MoS_2_ NFs-Ppy NPs under NIR exposure. RAW 264.7 cells treated with LPS combined with ALG and SrR exhibited an increased presence of dead cells compared to those treated with LPS alone. Notably, cells treated with ALG@SrR-MoS_2_ NFs-Ppy NPs demonstrated a decreased rate of cell death compared to the aforementioned groups. Conversely, cells exposed to ALG@SrR-MoS_2_ NFs-Ppy NPs exhibited a slight increase in apoptotic markers, but the majority of cells remained viable. Further analysis of green and red fluorescence intensities indicated a decline in apoptosis within the groups treated with ALG@SrR-MoS_2_ NFs-Ppy NPs, especially under NIR irradiation. These observations imply that the phototherapeutic effects of may enhance cell survival [73, 74].

**Fig. 5:**
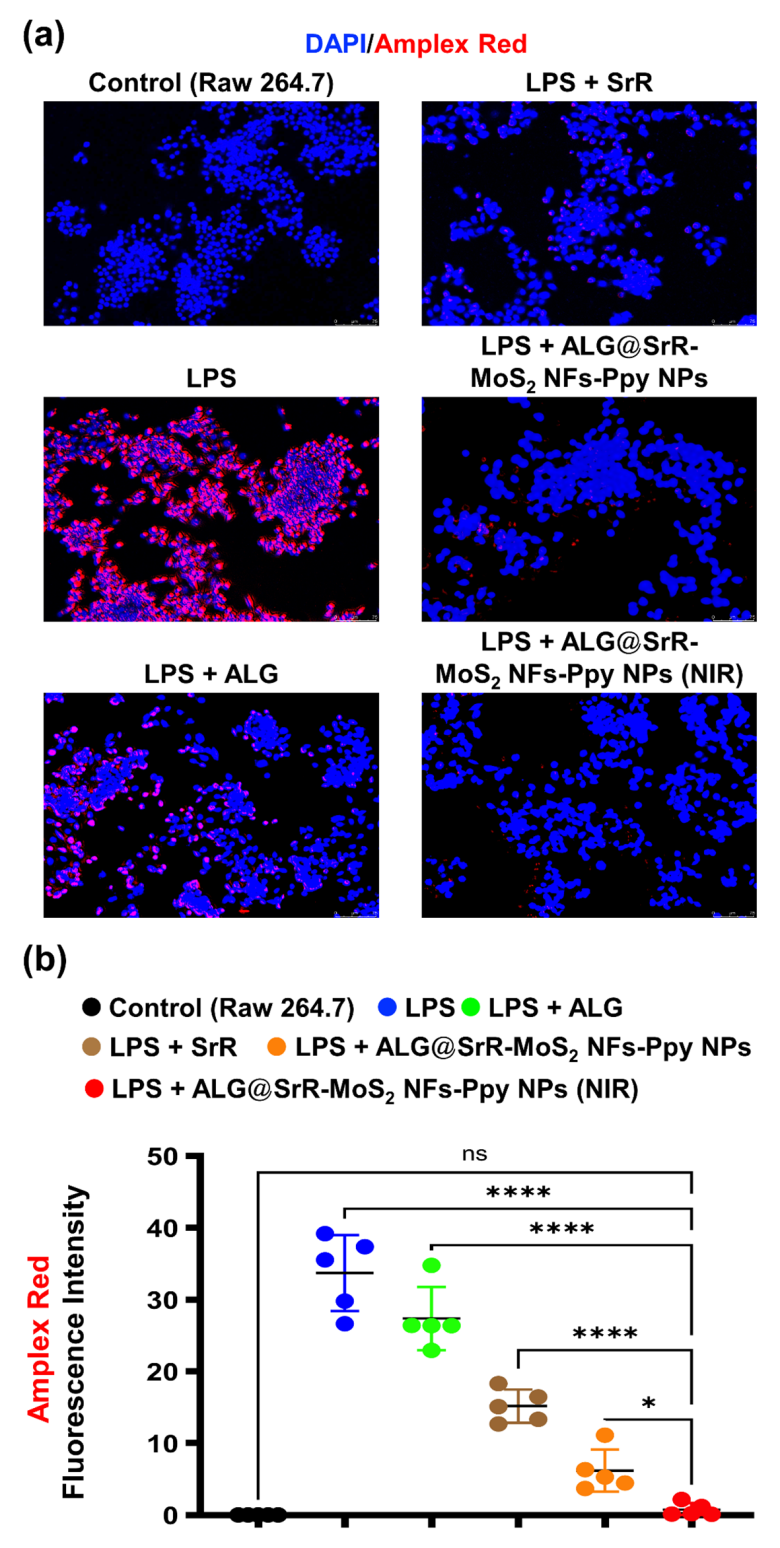
In vitro investigation of the cellular reactive oxygen species (ROS) levels in RAW 264.7 cells. (**a**, **b**) Data depicting levels of intracellular ROS after 24 h of incubation with different formulations, with a subset of cells stimulated by lipopolysaccharide (LPS). An Amplex red fluorescent probe was used for ROS quantification. Cells treated with LPS exhibited higher ROS levels, while those treated with the hydrogel formulations show reduced ROS, particularly in the ALG@SrR-MoS_2_ NF-Ppy NP + NIR group, which demonstrated a significant reduction in ROS (*p* < 0.01) after LPS stimulation, underscoring the potential anti-inflammatory effects of the phototherapeutic components. These data confirmed the protective effects of the ALG@SrR-MoS_2_ NF-Ppy NP hydrogel against cellular stress and highlighted the synergistic benefits of NIR irradiation in reducing inflammatory markers in macrophages. The study’s quantitative data are presented as the average ± standard deviation (SD) of a minimum of three replicate experiments (*n* ≥ 3). We used the two-way analysis of variance (ANOVA) to determine the statistical significance among several groups. GraphPad Prism software vers. 5.04 for Windows was used to do this analysis (Dotmatics, Boston, MA, USA). Thresholds of statistical significance were set to *p* < 0.05 (*), *p* < 0.01 (**), *p* < 0.001 (***), and *p* < 0.0001 (****)

Figure 5a and b present ROS levels in RAW 264.7 cells incubated with various formulations (ALG, SrR, ALG@SrR-MoS_2_ NFs-Ppy NPs, ALG@SrR-MoS_2_NFs-Ppy NPs + NIR) over 24 h, with subgroups subjected to LPS stimulation. ROS were assessed using an Amplex red fluorescent probe, a methodical approach for quantifying cellular ROS concentrations. Results indicated a marked elevation of ROS in cells subjected to LPS, emphasizing the inflammatory response elicited by the stimulus [75, 76].

A comparative analysis revealed that following LPS induction, intracellular ROS levels significantly varied among groups. Notably, in samples containing ALG, SrR, and ALG@SrR-MoS_2_ NFs-Ppy NPs, ROS levels were reduced compared to those in the group stimulated with only LPS. Cells that showed elevated ROS were limited to only those treated with LPS or LPS + ALG. Cells treated with the formulation of ALG@SrR-MoS_2_ NFs-Ppy NPs + NIR revealed negligible ROS levels. This further proves the inflammatory response elicited by the stimulus. This observation could be reflective of the inherent anti-inflammatory attributes of SrR and ALG, as delineated in previous research [29, 44, 49, 77].

Under LPS stimulation, the subgroup treated with ALG@SrR-MoS_2_ NFs-Ppy NPs in conjunction with NIR exposure demonstrated a substantial decrement in ROS levels (*p* < 0.05), suggesting a synergistic anti-inflammatory effect. This effect was potentially underpinned by photothermally induced mild hyperthermia [29] and electrical stimulation [43], attributable to the photoresponsive nature of the ALG@SrR-MoS_2_ NFs-Ppy NPs. Thus, the photothermal and photoelectric properties of these NPs may play a significant role in mitigating inflammatory responses at the cellular level, as evidenced by the pronounced reduction in ROS of treated cells. These NPs possess photothermal and photoelectric properties, which likely contributed to mitigating inflammatory responses at the cellular level, as evidenced by the pronounced reduction in ROS levels observed in treated cells. This finding underscores the potential of nanomaterial-based approaches, particularly those utilizing ALG@SrR-MoS_2_ NFs-Ppy NPs, in modulating inflammatory processes and highlights the importance of exploring their photothermal and photoelectric properties for therapeutic applications in inflammation-related disorders.

**Fig. 6:**
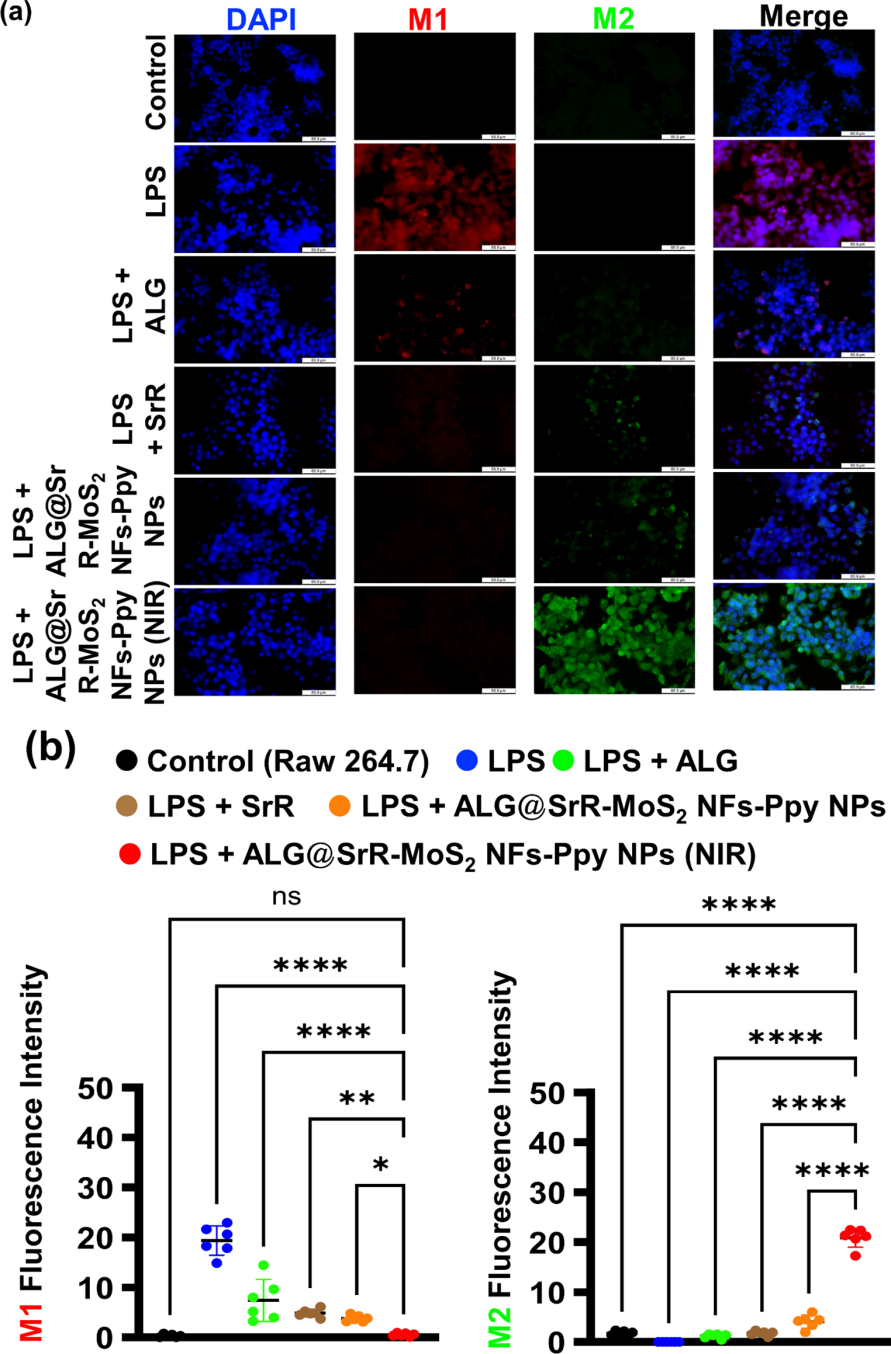
In vitro investigation of the cellular immunomodulation in RAW 264.7 cells. (**a**) Fluorescent labeling microscopic data and (**b**) analysis of macrophage polarization. LPS-treated RAW 264.7 cells exhibited increased M1 phenotype markers (CD86, red fluorescence) and decreased M2 markers (CD206, green fluorescence). Treatment with ALG@SrR-MoS2 NFs-Ppy NPs + NIR resulted in a notable decrease in M1 marker expression and an increase in M2 marker expression, compared to groups of RAW 264.7 cells + LPS, RAW 264.7 cells + LPS + ALG, RAW 264.7 cells + LPS + SrR, and RAW 264.7 cells + LPS + ALG@SrR-MoS_2_ NFs-Ppy NPs, indicating a shift towards anti-inflammatory M2 polarization. The study’s quantitative data are presented as the average ± standard deviation (SD) of a minimum of three replicate experiments (*n* ≥ 3). We used the two-way analysis of variance (ANOVA) to determine the statistical significance among several groups. GraphPad Prism software vers. 5.04 for Windows was used to do this analysis (Dotmatics, Boston, MA, USA). Thresholds of statistical significance were set to *p* < 0.05 (*), *p* < 0.01 (**), *p* < 0.001 (***), and *p* < 0.0001 (****)

The study further explored the influence of ALG@SrR-MoS_2_ NFs-Ppy NPs on macrophage phenotype reprogramming. CD86 (indicative of the M1 proinflammatory phenotype) and CD206 (representative of the M2 anti-inflammatory phenotype) fluorescent markers were used to evaluate the in vitro polarization of RAW264.7 cells. Figure 6a and b show that LPS-stimulated cells demonstrated higher M1-related red fluorescence and reduced M2-related green fluorescence compared to control cells. Remarkably, LPS-stimulated cells treated with ALG@SrR-MoS_2_ NFs-Ppy NPs + NIR showed a significant reduction in M1 red fluorescence and an increase in M2 green fluorescence, compared to the groups of RAW 264.7 cells + LPS, RAW 264.7 cells + LPS + ALG, RAW 264.7 cells + LPS + SrR, and RAW 264.7 cells + LPS + ALG@SrR-MoS_2_ NFs-Ppy NPs, suggesting a shift towards the M2 phenotype. This observed shift towards the M2 phenotype in response to NP treatment and NIR exposure suggests a potential modulatory effect on macrophage polarization, offering a promising avenue for interventions that can mitigate inflammatory responses and promote a pro-resolving environment. These findings provide valuable insights into the immunomodulatory properties of the NPs, adding a layer of complexity to their potential applications in addressing inflammatory conditions and macrophage-related disorders.

These findings indicate that the phototherapeutic application of ALG@SrR-MoS_2_ NFs-Ppy NPs can effectively modulate macrophage polarization. Previous studies supported the notion that mild hyperthermia and electric stimulation can influence macrophage polarization by exerting antioxidant and anti-inflammatory effects [78, 79]. Drawing from those precedents, it was inferred that the phototherapeutic effects of ALG@SrR-MoS_2_ NFs-Ppy NPs significantly contributed to the polarization balance between M1 and M2 macrophages, which may have important implications for therapeutic strategies targeting inflammatory conditions. The observed modulation of macrophage polarization by the phototherapeutic application of ALG@SrR-MoS_2_ NFs-Ppy NPs highlights the potential of these NPs in influencing immune responses. Previous research suggested that mild hyperthermia and electrical stimulation can impact macrophage polarization, resulting in antioxidant and anti-inflammatory effects. Leveraging this knowledge, it can be inferred that the phototherapeutic effects of ALG@SrR-MoS_2_ NFs-Ppy NPs may play a significant role in balancing the polarization between M1 and M2 macrophages. This balance is crucial in regulating inflammatory processes, and the ability of these NPs to modulate it could hold promising implications for therapeutic strategies targeting inflammatory conditions. By promoting a shift towards the anti-inflammatory M2 phenotype while inhibiting the proinflammatory M1 phenotype, ALG@SrR-MoS_2_ NFs-Ppy NPs offer a potential avenue for the development of novel therapies aimed at mitigating inflammatory disorders. Further exploration of the underlying mechanisms and therapeutic potential of these NPs in modulating macrophage polarization could pave the way for more-effective treatment approaches for inflammatory diseases.

**Fig. 7:**
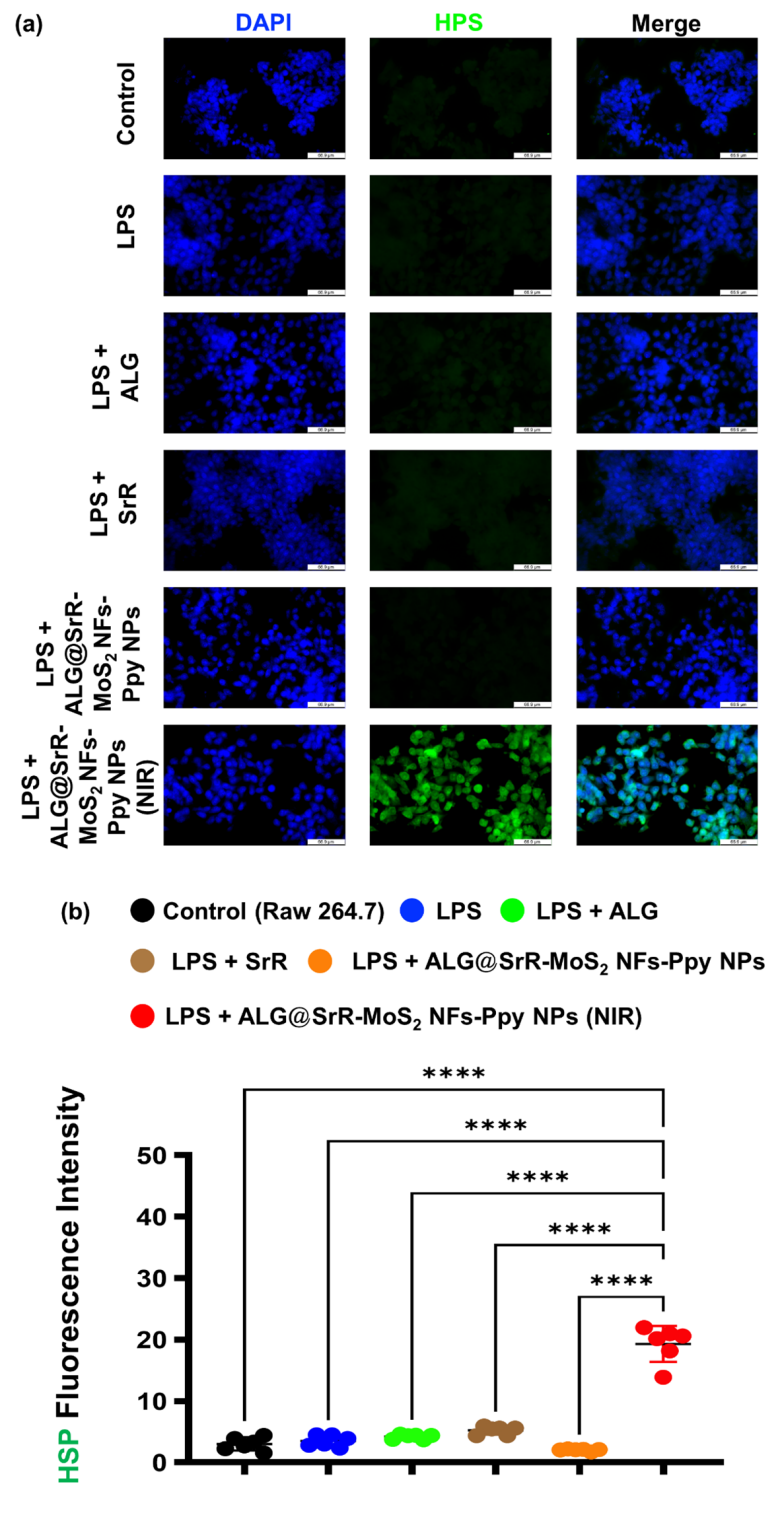
In vitro investigation of the cellular heat shock protein (HSP) levels in RAW 264.7 cells. (**a**) Microscopic and (**b**) quantitative evaluation of HSP expression by RAW 264.7 cells. Cells treated with LPS and ALG@SrR-MoS2 NFs-Ppy NPs + NIR demonstrated enhanced HSP expression compared to groups of RAW 264.7 cells + LPS, RAW 264.7 cells + LPS + ALG, RAW 264.7 cells + LPS + SrR, and RAW 264.7 cells + LPS + ALG@SrR-MoS_2_ NFs-Ppy NPs suggesting a protective effect against cellular stress. The study’s quantitative data are presented as the average ± standard deviation (SD) of a minimum of three replicate experiments (*n* ≥ 3). We used the two-way analysis of variance (ANOVA) to determine the statistical significance among several groups. GraphPad Prism software vers. 5.04 for Windows was used to do this analysis (Dotmatics, Boston, MA, USA). Thresholds of statistical significance were set to *p* < 0.05 (*), *p* < 0.01 (**), *p* < 0.001 (***), and *p* < 0.0001 (****)

HSPs are known to play pivotal roles in protecting organs from damage by ensuring the continuous synthesis and proper folding of proteins, facilitating the repair of damaged proteins, and enhancing the recovery of injured organs [49]. Microscopic observations revealed that RAW 264.7 cells subjected to LPS stress and treated with ALG@SrR-MoS_2_ NFs-Ppy NPs + NIR displayed elevated levels of cellular HSP expressions compared to the groups of RAW 264.7 cells + LPS, RAW 264.7 cells + LPS + ALG, RAW 264.7 cells + LPS + SrR, and RAW 264.7 cells + LPS + ALG@SrR-MoS_2_ NFs-Ppy NPs, as illustrated in Fig. 7a and b. The presence of Ppy within the ALG@SrR-MoS_2_ NFs-Ppy NPs was hypothesized to have stimulated the production of HSPs, possibly due to its photothermal characteristics. This finding underscores the importance of NPs in influencing cellular responses to stress, particularly through augmentation of HSPs, known for their essential functions in protein synthesis and folding, and cellular repair. Results of this study shed light on potential therapeutic applications of these NPs in enhancing cellular resilience and promoting recovery from stress-induced damage, offering valuable insights for future biomedical interventions. This implies that the NPs may modulate cellular stress responses, potentially offering therapeutic benefits in mitigating cellular damage and promoting resilience under stressful conditions. Further investigations into the molecular mechanisms underlying the observed increase in HSP expression and the therapeutic potential of these NPs in disease models characterized by cellular stress are warranted. These findings highlight the promising role of nanomaterial-based approaches in manipulating cellular stress responses for therapeutic purposes.

### In vivo assessments of biodistribution and behavioral tests

**Fig. 8:**
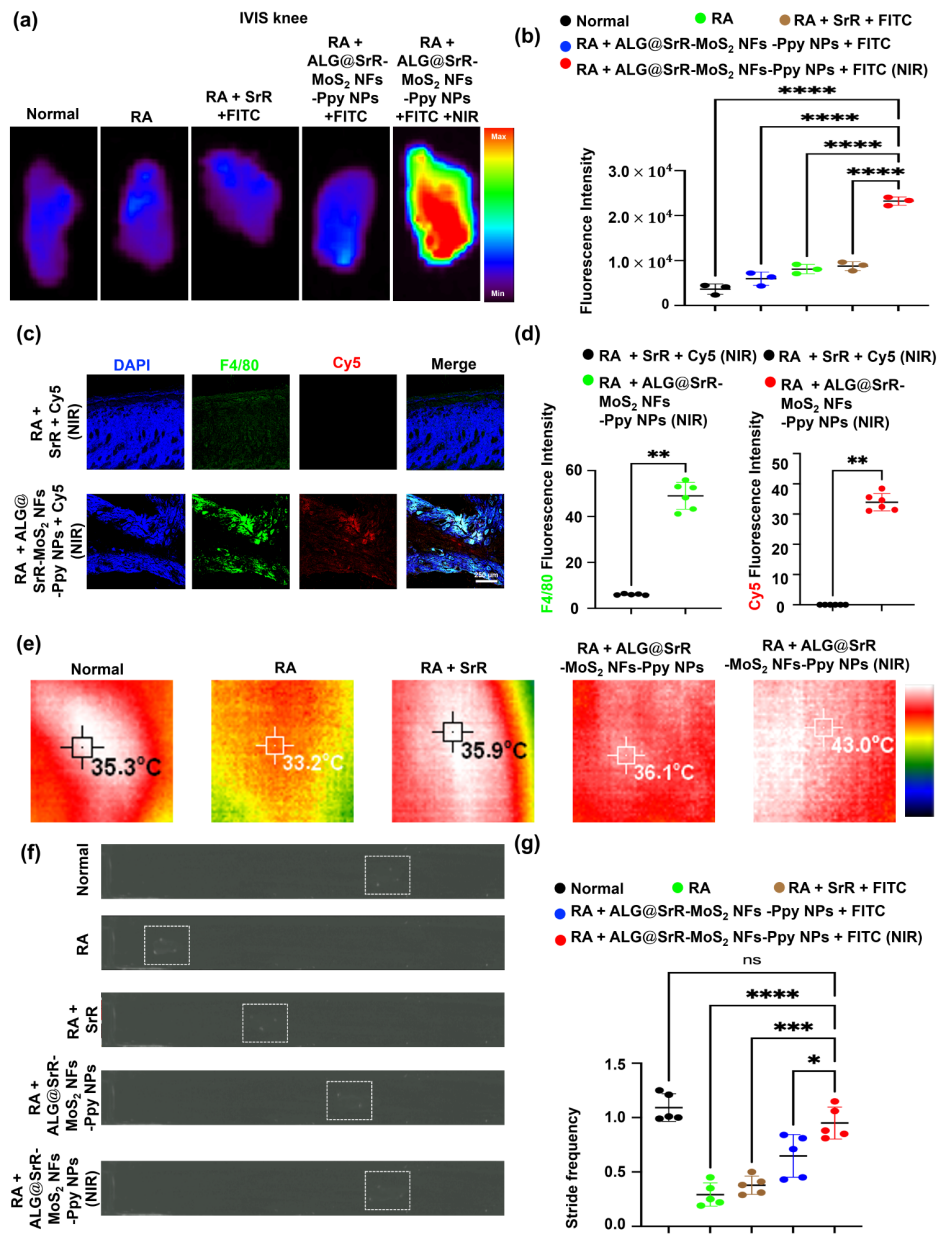
Evaluation of drug penetration, immune response, and functional recovery in rheumatoid arthritis (RA) mice treated with phototherapeutic hydrogels. (**a**, **b**) Fluorescein isothiocyanate (FITC) fluorescence imaging and analysis demonstrate the biodistribution of strontium ranelate (SrR) + FITC and SrR incorporated into alginate-based hydrogels containing molybdenum disulfide nanoflowers and polypyrrole nanoparticles (ALG@SrR-MoS_2_ NFs-Ppy NPs) containing FITC, with and without near infrared (NIR) treatment. Images reveal that the ALG@SrR-MoS_2_ NF-Ppy NP + NIR group achieved significant fluorescence in the joint cavity, indicating enhanced drug delivery. (**c**, **d**) Microscopic and quantitative analyses of Cy5 skin penetration and macrophage targeting in RA mice treated with SrR + Cy5 + NIR versus ALG@SrR-MoS_2_ NFs-Ppy NPs containing Cy5 + NIR. The latter group exhibited increased Cy5 fluorescence and macrophage accumulation, underscoring the targeted delivery and anti-inflammatory potential of the hydrogel. (**e**) Thermal imaging revealed that the ALG@SrR-MoS_2_ NFs-Ppy NPs exhibited a pronounced photothermal effect when subjected to NIR irradiation. (**f**, **g**) A gait analysis was used to measure the severity of arthritis and functional recovery. The highest stride frequency values were observed in the healthy control group, while the lowest were in the untreated RA group. Mice treated with ALG@SrR-MoS_2_ NFs-Ppy NPs + NIR showed an improved stride frequency, indicating alleviation of arthritic symptoms and enhanced mobility. These findings collectively demonstrated the profound impact of ALG@SrR-MoS_2_ NF-Ppy NP + NIR treatment on drug penetration, immune modulation, and functional improvement in this RA mouse model. The study’s quantitative data are presented as the average ± standard deviation (SD) of a minimum of three replicate experiments (*n* ≥ 3). We used the two-way analysis of variance (ANOVA) to determine the statistical significance among several groups. GraphPad Prism software vers. 5.04 for Windows was used to do this analysis (Dotmatics, Boston, MA, USA). The Student’s t-test was used for the statistical analysis of Fig. 8d. Thresholds of statistical significance were set to *p* < 0.05 (*), *p* < 0.01 (**), *p* < 0.001 (***), and *p* < 0.0001 (****)

This study emphasized the significant enhancement in drug penetration achieved using the composite phototherapeutic ALG@SrR-MoS_2_ NFs-Ppy NPs, particularly in the context of RA. An experimental RA model was induced in mice using zymosan, and results of the drug biodistribution (knee) are illustrated in Fig. 8a and b. In RA mice treated with SrR + FITC, minimal FITC fluorescence accumulation was noted in the joint cavity, indicating a limited penetration capacity of the free SrR + FITC formulation. Most notably, the group of RA mice treated with ALG@SrR-MoS_2_ NFs-Ppy NPs containing FITC + NIR demonstrated superior penetration ability within the RA joint cavity, with the most intense fluorescence signals observed. This result aligns with existing research indicating that hyperthermic conditions applied to the skin can significantly enhance drug delivery and facilitate the penetration of molecules [80]. Mild hyperthermia is also associated with an induced immune effect [81]. The enhancement of drug delivery through the application of hyperthermic conditions to the skin has been a subject of interest in transdermal drug delivery research. Hyperthermia, or the elevation of tissue temperature, can enhance the permeability of the skin by several mechanisms, making it a valuable technique for improving the efficacy of topical and transdermal therapeutic systems. Hyperthermia was shown to increase the fluidity of lipid bilayers in the stratum corneum, the outermost layer of the skin, which acts as the primary barrier to drug penetration. As the temperature increases, the lipid matrix softens, allowing larger molecules to more readily penetrate. Previously published research discussed how controlled heating can disrupt the highly ordered structure of skin lipids, enhancing permeability [82]. An elevated skin temperature increases microvascular blood flow, which can facilitate the removal of absorbed substances from the dermis, preventing their back-diffusion and promoting deeper penetration. This effect was highlighted in [83], which discussed how increased blood flow at elevated temperatures enhances the systemic absorption of drugs. According to Fick’s laws of diffusion, the diffusion coefficient increases with temperature. The increased kinetic energy at higher temperatures results in more-rapid movement of drug molecules, as detailed in one study [84]. Hyperthermia often leads to increased sweating, which in turn increases skin hydration. Hydrated skin has a less-resistant stratum corneum, which significantly enhances drug penetration, a concept explored in [85]. Several commercial transdermal systems incorporate heat to improve drug delivery. For example, heat patches that increase local skin temperature are used to enhance the absorption of analgesics or anti-inflammatory agents, as described in [86]. Those studies and mechanisms highlight the scientific basis for using hyperthermia as a method to enhance the percutaneous delivery of drugs. By understanding and applying these principles, more effective and efficient transdermal drug delivery systems can be developed, particularly for drugs with poor skin permeability.

Of particular significance is the superior penetration ability observed in the group of RA mice treated with ALG@SrR-MoS_2_ NFs-Ppy NPs containing FITC and subjected to NIR exposure, demonstrating the most intense fluorescence signals within the joint cavity. This finding is consistent with previous research indicating that hyperthermic conditions induced by NIR exposure can markedly enhance drug delivery and facilitate molecule penetration [87] Furthermore, mild hyperthermia is associated with an induced immune effect. These results suggest that the phototherapeutic properties of ALG@SrR-MoS_2_ NFs-Ppy NPs, particularly when combined with NIR exposure, offer a promising approach to enhance drug penetration in RA treatment. Leveraging hyperthermic conditions not only improves drug delivery but also potentially augments the immune response, providing a multifaceted therapeutic strategy for addressing inflammatory conditions such as RA. Further exploration of the underlying mechanisms and optimization of NIR exposure parameters could lead to the development of more-effective targeted drug delivery systems for inflammatory diseases.

Further microscopic and skin quantitative analyses, as shown in Fig. 8c and d, suggest that RA mice treated with SrR + a fluorescent probe (Cy5) + NIR exhibited minimal Cy5 fluorescence penetration and low macrophage accumulation. In stark contrast, the skin of RA mice treated with ALG@SrR-MoS_2_ NFs-Ppy NPs containing Cy5 + NIR showed significantly increased Cy5 fluorescence and a marked increase in macrophage accumulation. The effective penetration and immune cell targeting were primarily attributed to the photothermally responsive component of ALG@SrR-MoS_2_ NFs-Ppy NPs preferentially concentrating on immune cells in inflamed joints. The microscopic and quantitative analyses revealed distinct outcomes in the effectiveness of different treatment modalities for RA. While RA mice treated with SrR and NIR exposure exhibited limited penetration of the fluorescent probe (Cy5) and low macrophage accumulation, those treated with ALG@SrR-MoS_2_ NFs-Ppy NPs containing Cy5 and subjected to NIR exposure demonstrated significantly increased Cy5 fluorescence and marked macrophage accumulation within inflamed joints. These findings underscore the importance of the photothermally responsive component in facilitating effective penetration and immune cell targeting, highlighting the potential of nanomaterial-based approaches for enhancing precision and efficacy in RA therapy. Further elucidation of underlying mechanisms and translational studies are warranted to optimize treatment strategies and realize personalized therapies for inflammatory diseases. The observed contrast between the treatment groups highlights the potential of nanomaterial-based approaches in improving the precision and effectiveness of therapeutic interventions for inflammatory diseases like RA. Additionally, exploring the translational potential of these findings in clinical settings could pave the way for the development of personalized and targeted therapies for patients suffering from RA and other inflammatory conditions.

The thermal imaging demonstrated that the application of NIR irradiation to the ALG@SrR-MoS_2_ NFs-Ppy NPs resulted in a significant increase in temperature, reaching mild-hyperthermic levels, according to the thermal image data (Fig. 8e). This indicates an effective photothermal response, particularly when compared to the normal, RA, RA + SrR, and RA + ALG@SrR-MoS_2_ NFs-Ppy NPs groups, where such a temperature elevation was not observed.

Additionally, the severity of arthritis was evaluated using a gait analysis, with results presented in Fig. 8f and g. The gait analysis revealed that the normal healthy group exhibited the highest stride frequency values, while the untreated arthritis group showed the lowest. Importantly, arthritic mice treated with ALG@SrR-MoS_2_ NFs-Ppy NPs + NIR displayed an increase in stride frequency compared to their untreated counterparts, as well as those treated with SrR alone or with ALG@SrR-MoS_2_ NFs-Ppy NPs, highlighting the therapeutic potential of this phototherapeutic approach in mitigating arthritis symptoms.

### In vivo biosafety, therapeutic, and radiographic evaluations

**Fig. 9:**
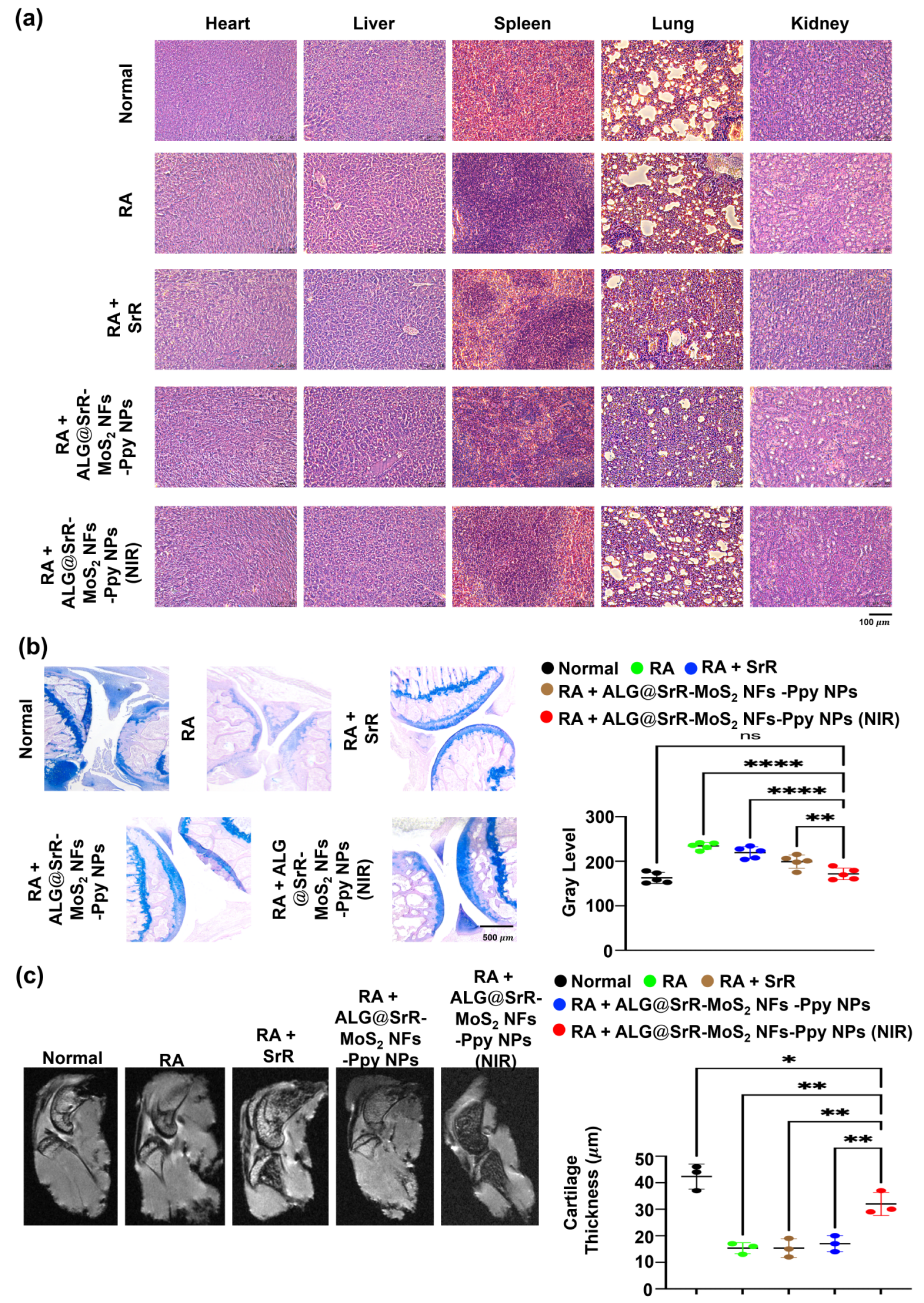
Toxicity assessment, cartilage erosion analysis, and MRI evaluation in rheumatoid arthritis (RA) mice treated with strontium ranelate incorporated into alginate-based hydrogels containing molybdenum disulfide nanoflowers and polypyrrole nanoparticles (ALG@SrR-MoS_2_ NFs-Ppy NPs). (**a**) H&E staining of major organs (heart, liver, spleen, lungs, and kidneys) from different treatment groups, including ALG@SrR-MoS_2_ NFs-Ppy NPs with near infrared (NIR) therapy. Staining results indicated no significant morphological changes in these organs compared to the sham group, suggesting minimal systemic toxicity. (**b**) Alcian blue (AB) staining of knee joint cartilage in various groups. Light-blue staining in untreated and SrR-treated arthritic groups indicates potential cartilage damage, while intense staining in the ALG@SrR-MoS_2_ NF-Ppy NP + NIR group resembles healthy cartilage, indicating effective protection against cartilage erosion. (**c**) T2-weighted MRI images were used to assess cartilage thickness and tissue integrity in RA knees. This comprehensive analysis underscores the safety and efficacy of ALG@SrR-MoS_2_ NFs-Ppy NPs, particularly when combined with NIR therapy, in mitigating arthritis symptoms and progression, while maintaining a favorable safety profile. Quantitative data in this study are expressed as the mean ± standard deviation of at least three experiments done in triplicate (*n* ≥ 3). To ascertain the statistical significance between multiple groups, we employed the nonparametric Kruskal-Wallis ANOVA. This analysis was conducted using GraphPad Prism software vers. 5.04 for Windows. Statistical significance thresholds were established at *p* values of < 0.05 (*), < 0.01 (**), < 0.001 (***), and 0.0001 (****)

The potential of ALG@SrR-MoS_2_ NFs-Ppy NPs in clinical applications was further assessed by examining their toxicity toward major organs in mice. H&E staining was employed to evaluate the tissue morphology of the heart, liver, spleen, lungs, and kidneys across different treatment groups, as shown in Fig. 9a. A comparative analysis with the sham group revealed no significant morphological alterations in these organs, suggesting that ALG@SrR-MoS_2_ NFs-Ppy NPs, in conjunction with NIR therapy, exhibited no overt toxicity, while the RA + SrR group and RA + ALG@SrR-MoS_2_ NF-Ppy NP-treated group indicated obvious alveolar wall thickening. RA causes diffuse alveolar hemorrhage and bronchial wall thickening [88], and anti-inflammatory rituximab alleviates this manifestation. These findings indicated that ALG@SrR-MoS_2_ NFs-Ppy NPs possessed excellent in vivo biosafety, avoiding alveolar wall thickening through potential anti-inflammation, and positioning them as promising candidates for targeted drug delivery in clinical settings.

Cartilage erosion was investigated using Alcian blue (AB) staining, as depicted in Fig. 9b. Staining results highlighted that cartilage in the untreated arthritic groups, those receiving only SrR, and groups treated with ALG@SrR-MoS_2_ NFs-Ppy NPs showed light-blue staining, indicative of potential cartilage damage. In contrast, articular cartilage in arthritic mice treated with ALG@SrR-MoS_2_ NFs-Ppy NPs + NIR exhibited more-intense staining, resembling healthy cartilage.

MRI, particularly T2-weighted imaging, plays a crucial role in biomedical research, especially for detecting and quantifying inflammatory involvement in RA, as previously noted [89–91]. T2 MRI’s ability to precisely assess tissue cartilage thickness makes it a potential reference tool in RA diagnosis and management.

MRI images of RA knees, as shown in Fig. 9c, demonstrated marked periarticular cartilage thickness in the lower level, compared to a normal healthy condition. Conversely, higher cartilage thickness levels and well-preserved knee anatomy were observed in healthy mice and arthritic mice treated with ALG@SrR-MoS_2_ NFs-Ppy NPs + NIR. These outcomes suggested that ALG@SrR-MoS_2_ NF-Ppy NP + NIR treatment can effectively alleviate arthritis symptoms and decelerate disease progression.

### In vivo histological test

**Fig. 10:**
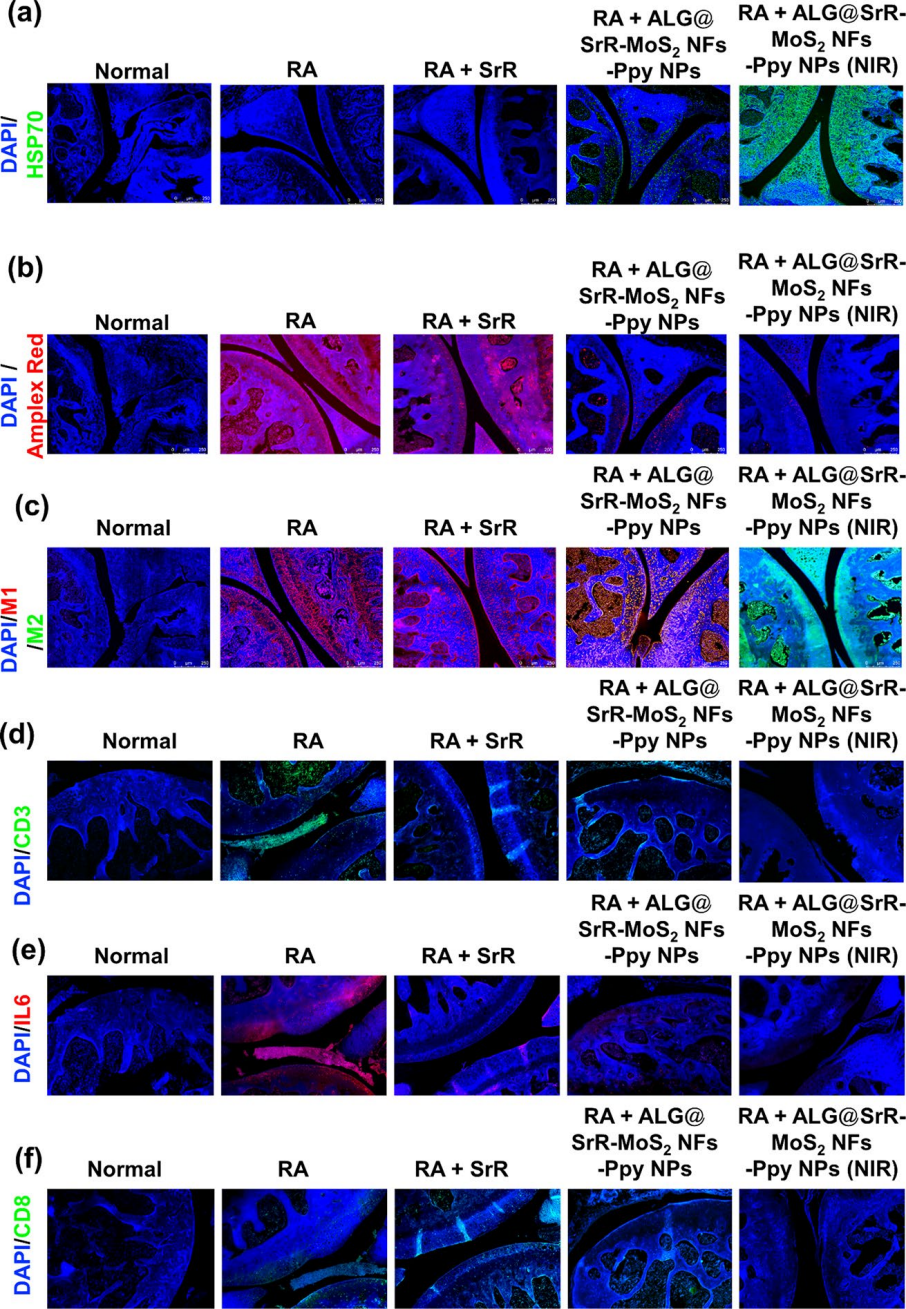
Assessment of heat shock protein (HSP) levels, reactive oxygen species (ROS), interleukin (IL)-6, T-cell activity (CD8^+^), T-cell activity (CD3^+^), and macrophage polarization in rheumatoid arthritis (RA) mice treated with strontium ranelate incorporated into alginate-based hydrogel containing molybdenum disulfide nanoflowers and polypyrrole nanoparticles (ALG@SrR-MoS_2_ NFs-Ppy NPs). (**a**) HSP expression in cartilage tissues, showing elevated HSP levels in the ALG@SrR-MoS_2_ NFs-Ppy NPs + NIR group, suggesting enhanced protective effects on cartilage lesions and potential applications in tissue engineering. (**b**) ROS detection using an Amplex red assay. Elevated ROS levels in the arthritis control group indicated increased inflammation, while a significant reduction in ROS was observed in groups treated with ALG@SrR-MoS_2_ NFs-Ppy NPs, especially with additional NIR therapy, indicating effective anti-inflammatory action. (**c**) Immunofluorescence (IF) staining for the macrophage polarization analysis in synovial tissues. Elevated CD86 expression (an M1 macrophage marker) in the RA control and treatment groups without NIR indicates a predominance of proinflammatory M1 macrophages. In contrast, the ALG@SrR-MoS_2_ NF-Ppy NP + NIR group showed reduced CD86 expression and increased CD206 expression (an M2 macrophage marker), highlighting effective modulation towards anti-inflammatory M2 macrophages. (**d**) Cellular expressions of CD3^+^ and (**e**) IL-6 in the treated groups. (**f**) CD8^+^ in the treated groups. These findings comprehensively demonstrate the multifaceted therapeutic impacts of ALG@SrR-MoS_2_ NF-Ppy NP + NIR treatment in an RA model, encompassing enhanced HSP expression, reduced ROS levels, and favorable macrophage polarization, thereby substantiating its potential for clinical application in RA management.

In this study, increased HSP70 levels in cartilage were observed in the ALG@SrR-MoS_2_ NFs-Ppy NPs + NIR group (Fig. 10a), indicating a potential protective role of HSPs in cartilage lesions, relevant to tissue engineering applications. RA is a systemic chronic inflammatory disease, characterized by synovial hyperproliferation, macrophage infiltration, and dysregulated autoimmune responses [92, 93]. Photothermal Ppy NPs were identified as a versatile phototherapeutic biomaterial capable of exerting regulatory effects on various immunocytes. Given the aromatic structure of Ppy, its photothermal responsiveness may induce HSP upregulation, positioning Ppy NPs as a promising candidate for arthritis treatment [29, 94]. HSPs play critical roles in stress recovery, either by repairing or degrading damaged proteins, thereby restoring protein homeostasis and enhancing cell survival [95, 96].

Furthermore, cellular ROS levels, indicative of local biological inflammation, were assessed using an Amplex red assay (Fig. 10b). The arthritis control group exhibited significantly higher ROS levels in synovial tissues compared to the normal healthy group. After treatment with the hydrogel (ALG@SrR-MoS_2_ NFs-Ppy NPs + NIR), ROS expression levels were notably reduced, compared to the RA + SrR or RA untreated groups. In particular, the ALG@SrR-MoS_2_ NF-Ppy NP + NIR group showed a substantial decrease in ROS levels, aligning closely with those of the normal healthy group.

Macrophage polarization in synovial tissues was evaluated using IF staining (Fig. 10c). In the RA control, RA + SrR, and RA + ALG@SrR-MoS_2_ NF-Ppy NP groups, elevated CD86 expression (an M1 macrophage marker) indicated an abundance of M1 macrophages in synovial organs. In contrast, the ALG@SrR-MoS_2_ NF-Ppy NP + NIR group displayed significantly reduced CD86 fluorescence, suggesting effective in vivo suppression of M1 macrophage polarization. Additionally, CD206 expression (an M2 macrophage marker) in the ALG@SrR-MoS_2_ NF-Ppy NP + NIR group markedly differed from that in the arthritis and treated groups (RA, RA + SrR, RA + ALG@SrR-MoS_2_ NFs-Ppy NPs), indicating enhanced polarization towards M2 macrophages. The study revealed that ALG@SrR-MoS_2_ NF-Ppy NP + NIR treatment can alleviate joint inflammation primarily by suppressing M1 macrophage polarization and promoting M2 macrophage polarization. This synergistic therapeutic approach was evaluated in vivo using a rodent model of RA, focusing on inflammatory symptoms, articular cartilage repair, M2 macrophage polarization, inflammation suppression, HSP expressions, and cartilage extracellular matrix degeneration.

Previous research has indicated a rise in CD3^+^ T cells in individuals with RA [97]. In the case of rheumatoid arthritis (RA), patients typically show heightened levels of interleukin-6 (IL-6). Upon microscopic examination, it was observed that animals with RA displayed significantly elevated CD3^+^ T cell counts, as depicted in Fig. 10d. Notably, treatment regimens involving RA, RA + SrR, and RA + ALG@SrR-MoS_2_ NFs-Ppy NPs exhibited only marginal reductions in CD3^+^ T cell populations. Conversely, the group treated with RA + ALG@SrR-MoS_2_ NF-Ppy NP + NIR displayed a substantial decrease in CD3^+^ T cell expression. The findings underscore the importance of addressing CD3^+^ T cell activity in arthritis management and suggest that the RA + ALG@SrR-MoS2 NF-Ppy NP + NIR treatment approach holds promise for effectively modulating T cell responses in rheumatoid arthritis. Further investigation into the mechanisms underlying this observed reduction is warranted, potentially shedding light on novel therapeutic avenues for autoimmune disorders like RA. Similarly, these animals showed the most substantial levels of IL-6, as detailed in Fig. 10e. While the RA, RA + SrR, and RA + ALG@SrR-MoS_2_ NF-Ppy NP groups saw modest declines in IL-6 levels, NIR application in the RA + ALG@SrR-MoS_2_ NF-Ppy NP + NIR group led to a significant reduction in IL-6 expression. These findings suggest a potent anti-inflammatory response triggered by the phototherapeutic effects of the ALG@SrR-MoS_2_ NFs-Ppy NPs when combined with NIR treatment. Marked decreases in proinflammatory markers like CD8 + T cells and IL-6 in the NIR-treated group underscore the potential of integrating photothermal therapy in managing inflammatory conditions. Notably, NIR treatment seemed to enhance the hydrogel’s capacity to modulate immune responses, potentially offering a targeted approach to treat chronic inflammation in RA. This could have been due to the localized heat generated by NIR exposure, which may alter the cellular environment, reducing the activities of key inflammatory cells and cytokines. Further investigation into the cellular mechanisms affected by this treatment could provide deeper insights into its therapeutic benefits and optimize its clinical applications.

Previous studies demonstrated an increase in CD8 + T cells among osteoarthritic patients [98]. Patients with RA have elevated IL-6 levels [99]. Microscopic observations indicated that animals with RA exhibited the highest levels of CD8 + T cells, as shown in Fig. 10f. Treatment groups receiving RA, RA + SrR, and RA + ALG@SrR-MoS_2_ NFs-Ppy NPs experienced only a slight reduction in CD8 + T cell counts. In contrast, the RA + ALG@SrR-MoS_2_ NF-Ppy NP + NIR group exhibited a pronounced decrease in CD8 + T cell expression.

## Conclusions

RA is a chronic degenerative joint disease that has immense adverse effects on a patient’s quality of life. The lack of sufficient biodistribution makes therapeutic effects of numerous medication treatment approaches poor even after extensive research. In order to accomplish high-efficiency drug delivery, immunomodulation, anti-inflammation, and HSP amplification, functional phototherapeutic formulations have recently been investigated. Here, phototherapeutic polypyrrole NPs and MoS2 NFs, and ALG hybrid hydrogel-laden SrR were created for RA treatment. We employed this approach in order to assess phototherapeutic outcomes and targeted delivery in a synergistic manner. Due to photoresponsivity under NIR irradiation, this biomimetic formulation demonstrated notable cytocompatibility as well as immunomodulatory, anti-inflammatory, and HSP amplification capabilities in vitro. Significantly, in vivo investigations demonstrated that the phototherapeutic hydrogel drug delivery method achieved a superior therapeutic effect in potentially delaying the onset of RA. Therefore, by biophyical stimulation, and improved drug delivery, this unique hybrid hydrogel-coated phototherapeutic nanoformulation demonstrated significant therapeutic efficacy and can possibly be developed into a promising drug delivery platform for enhancing therapeutic benefits against RA.

This innovative approach leveraged NIR irradiation to activate photoresponsive elements, enhancing potential drug delivery, immunomodulation, and expressions of HSPs. While our in vitro and in vivo results demonstrated significant therapeutic efficacy, improving the onset delay of RA symptoms through localized biophyical stimulation, and targeted drug delivery, it is crucial to address some inherent limitations in the synthesis and applicability of this formulation. First, the synthesis of this composite material involves complex, multi-step procedures that might not easily scale up for commercial production. The precise control of the NP size, uniformity, and functionalization during synthesis is challenging and critical for the consistency of the therapeutic effects. Additionally, the stability of the NPs within the hydrogel matrix over time and under different physiological conditions remains a concern that could affect the long-term viability of this treatment approach.

Also, while the formulation shows promise in controlled laboratory settings, its real-world application could encounter several hurdles. The requirement for NIR equipment to activate the formulation may limit its use in settings without such specialized facilities. Moreover, the depth of NIR penetration and its uniformity can vary between individuals due to differences in tissue composition, potentially leading to inconsistent treatment outcomes. Furthermore, the potential for immunogenicity or unexpected biological responses to long-term exposure of these foreign materials in the human body has not been fully explored. This is a crucial aspect that requires thorough investigation to ensure the safety and efficacy of the treatment for long-term clinical use. Despite these challenges, the promising results of our phototherapeutic approach underscore its potential as a novel RA treatment. Future studies should aim to refine the synthesis processes for scalability, enhance the formulation’s stability, and further assess its safety profile. These steps are essential to transition from experimental to practical clinical applications, ultimately aiming to provide RA patients with a more-effective and less-invasive treatment option.

## Electronic supplementary material

Below is the link to the electronic supplementary material.

Supplementary Material 1

## Data Availability

The datasets used in this research study are available from the corresponding author upon reasonable request.
