# Supplementary material for: Alleviating rheumatoid arthritis with a photo-pharmacotherapeutic glycan-integrated nanogel complex for advanced percutaneous delivery
Source: J Nanobiotechnology. 2024 Oct 21;22:646. doi: 10.1186/s12951-024-02877-8 (PMC11492540; doi:10.1186/s12951-024-02877-8)
Supplement: Supplementary file 1 — Supplementary Material 1 [file 12951_2024_2877_MOESM1_ESM.docx]

**Supporting Information**


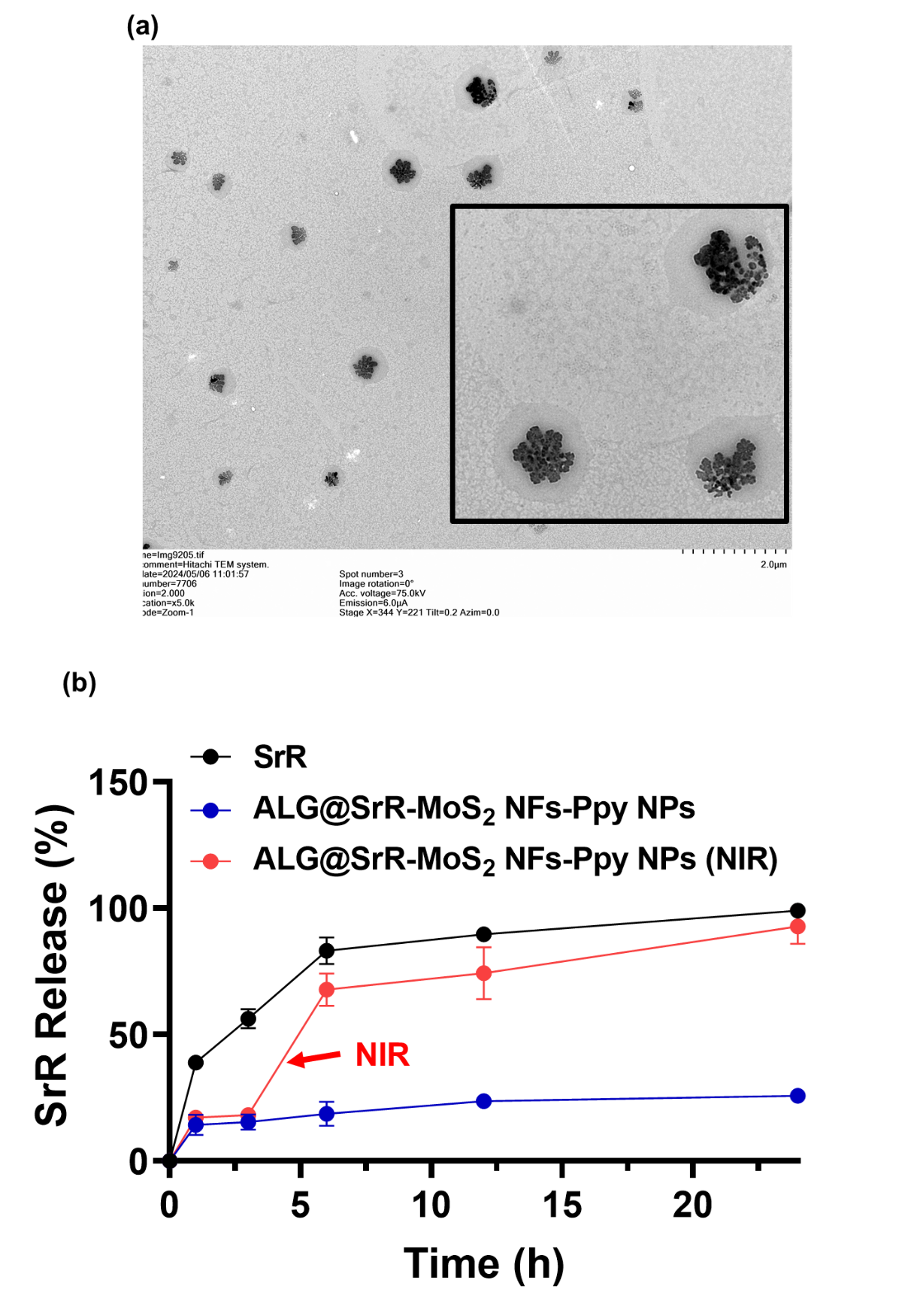


**Fig. S1**: (a) Transmission electron microscopic (TEM) analysis of the alginate incorporated into strontium ranelate molybdenum disulfide nanoflower polypyrrole nanoparticle (ALG@SrR-MoS_2_ NF-Ppy NP) composite. This figure showcases the polymeric filaments within the hydrogel matrix that encapsulate the MoS_2_ NFs and Ppy NPs. Encapsulation is critical for maintaining the structural integrity and enhancing the functional properties of the composite material. (b) Drug release kinetics of SrR from ALG@SrR-MoS_2_ NFs-Ppy NPs under photo-irradiation. This figure illustrates the increase in absorbance at 321 nm, indicating the release of SrR upon near infrared (NIR) exposure, reaching a saturation point at 24 h. The data highlight the rapid release with NIR exposure compared to minimal release without NIR. This demonstrates the precise control over drug release through photo-irradiation, allowing for adjustable and customizable release profiles.
